# Environmental correlates for tree occurrences, species distribution and richness on a high-elevation tropical island

**DOI:** 10.1093/aobpla/plv075

**Published:** 2015-07-10

**Authors:** Philippe Birnbaum, Thomas Ibanez, Robin Pouteau, Hervé Vandrot, Vanessa Hequet, Elodie Blanchard, Tanguy Jaffré

**Affiliations:** 1 CIRAD, UMR 51 AMAP, 34398 Montpellier, France; 2 Laboratory of Applied Botany and Plant Ecology, Institut Agronomique néo-Calédonien (IAC), Diversité biologique et fonctionnelle des écosystèmes terrestes, 98848 Noumea, New Caledonia; 3 Laboratory of Applied Botany and Plant Ecology, Institut de Recherche pour le Développement (IRD), UMR 123 AMAP, 98848 Noumea, New Caledonia

**Keywords:** Area effect, biodiversity hotspot, α-diversity, island, species richness, tropical mountains, ultramafic substrate

## Abstract

High-elevation tropical islands are ideally suited for examining the factors that determine species distribution, given the complex topographies and climatic gradients that create a wide variety of habitats within relatively small areas. New Caledonia, a megadiverse Pacific archipelago, has long focussed the attention of botanists working on the spatial and environmental ranges of specific groups, but few studies have embraced the entire tree flora of the archipelago. In this study we analyse the distribution of 702 native species of rainforest trees of New Caledonia, belonging to 195 genera and 80 families, along elevation and rainfall gradients on ultramafic (UM) and non-ultramafic (non-UM) substrates. We compiled four complementary data sources: (i) herbarium specimens, (ii) plots, (iii) photographs and (iv) observations, totalling 38 936 unique occurrences distributed across the main island. Compiled into a regular 1-min grid (1.852 × 1.852 km), this dataset covered ∼22 % of the island. The studied rainforest species exhibited high environmental tolerance; 56 % of them were not affiliated to a substrate type and they exhibited wide elevation (average 891 ± 332 m) and rainfall (average 2.2 ± 0.8 m year^−1^) ranges. Conversely their spatial distribution was highly aggregated, which suggests dispersal limitation. The observed species richness was driven mainly by the density of occurrences. However, at the highest elevations or rainfalls, and particularly on UM, the observed richness tends to be lower, independently of the sampling effort. The study highlights the imbalance of the dataset in favour of higher values of rainfall and of elevation. Projected onto a map, under-represented areas are a guide as to where future sampling efforts are most required to complete our understanding of rainforest tree species distribution.

## Abstract (precis)

This article focuses on the distribution of trees on a high-elevation tropical island of the New Caledonian archipelago. The aim was to determine how the variety of environments occurring on this island (in terms of elevation, rainfall, substrate and vegetation types) shapes the distribution of tree species. We analyzed the distribution of 702 native rainforest species through ca. 40,000 occurrence records and GIS environmental layers. Results showed that species exhibit high environmental tolerance while their distribution is spatially highly aggregated. We concluded that tree species distribution in New Caledonia is shaped by dispersal limitation rather than by environmental specialization.

## Introduction

Despite an ever increasing amount of data, the geographical distribution of most plant species remains incomplete and biased, particularly for the most diverse taxonomic groups and regions (Whittaker *et al*. 2005). This so-called Wallacean shortfall frequently originates from the difficulty of evaluating the distribution of species diversity across large, heterogeneous areas where biodiversity is high but collecting efforts have been insufficient or inadequately planned (Schmidt-Lebuhn *et al*. 2012). However, when correctly assessed and accounted for, sampling biases do not entirely prevent identification of the mechanistic determinants of species distribution, which remains a central question in ecology and biogeography (Hortal *et al*. 2007).

Many hypotheses have been proposed to explain the geographic variability of species diversity. It is widely accepted that species richness decreases poleward (Hillebrand 2004). However, the relationship between species richness and elevation is more complex and highly dependent on the organism being considered (Lomolino 2001; Nogués-Bravo *et al*. 2008; McCain and Grytnes 2010; Kessler *et al*. 2011).

Environmental conditions (air temperature, precipitation and solar radiation) change with elevation and the available area tends to decrease as elevation increases, due to the conical shape of mountains, thus affecting the total number of species (McCain 2007; Barry 2008). After accounting for the decreasing availability of area with increasing elevation, many organisms show a hump-shaped pattern of richness with elevation. In a meta-analysis involving 204 elevational transects, Rahbek (2005) found ∼80 % of hump-shaped richness patterns but only a small proportion of monotonic patterns. A number of explanations have been examined for these hump-shaped richness patterns (e.g. climate-derived productivity, source–sink dynamics, intermediate disturbance and mid-domain effect) but at this stage the dominant contributing factors remain unclear (McCain and Grytnes 2010).

Mountainous tropical islands are ideally suited for examining species distribution along complex environmental transects by virtue of their exaggerated climatic gradients and their complex topography. These combine to create a wide variety of habitats within a relatively small area. In addition to exhibiting steep environmental gradients, islands host biota with sharp variations in environmental tolerance and, paradoxically, harbour a greater proportion of narrow-range endemics when compared with continents (Carlquist 1974; Caujapé-Castells *et al*. 2010).

New Caledonia, an archipelago located in southwest Pacific Ocean, hosts a rich (more than 3200 species) and unique vascular flora (75 % of endemism) distributed within a remarkable mosaic of habitats (Morat *et al*. 2012). First considered as a vicariant Gondwanan refugium (Morat 1993), geologists have recently demonstrated the entire submersion by subduction before a final re-emersion of the main island ‘Grande Terre’ ∼37 My ago coated with a fragment of oceanic crust at the origin of the ultramafic (UM) substrates (Cluzel and Chiron 1998; Neall and Trewick 2008). The entire submersion thus argues for a secondary colonization origin for the entire New Caledonian flora. Sherwin Carlquist was one of the first to support this view and stated in his book ‘Island Biology’ that there is ‘no reason why New Caledonian flowering plants cannot be hypothesised to have arrived via long-distance dispersal’ (Carlquist 1974). Several phylogenetic studies have provided evidence supporting the thesis that the flora of New Caledonia indeed originates from recent (<37 My) long-distance dispersal colonizations and diversifications (Murienne *et al*. 2005; Grandcolas *et al*. 2008; Murienne 2009; Espeland and Murienne 2011; Pillon 2012).

Such biogeographical mysteries have long focussed the attention of local and international botanists over more fundamental studies of rainforest structure, floristic composition and ecology. Most earlier studies examining plant distribution patterns in New Caledonia have focussed on particular groups: endangered species (Herbert 2006; Kumar and Stohlgren 2009), emblematic species (Pintaud *et al*. 1999; Jaffré *et al*. 2010; Poncet *et al*. 2013), narrow-range endemic species (Wulff *et al*. 2013) or montane species (Nasi *et al*. 2002). To our knowledge, few studies have embraced the entire tree flora of the archipelago.

The few plot-based studies, that have investigated an exhaustive number of tree species, point to a consensus that the species assemblage is of similar interest to the unusual biogeographical history of the archipelago (e.g. Jaffré and Veillon 1990; Read *et al*. 2000; Ibanez *et al*. 2014). In particular, the New Caledonian rainforests seem to be characterized by very high stem density, low α-diversity (low local richness) and high β-diversity (high between-plot dissimilarity) without significant correlations with either elevation or rainfall (Grytnes and Felde 2014). However, it is fair to question whether the apparent independence of diversity from the environment arises from an unusually high environmental tolerance of the New Caledonian tree species or from a failure of experimental design to capture the species α-diversity (Grytnes and Felde 2014).

In this study, we used a new-occurrences dataset to test whether the environment is the main force driving New Caledonian rainforest tree species diversity and individual species distribution. In other words, does the species assemblage in rainforests reflect a deterministic model (i.e. an environment or niche-based one) or a stochastic model (i.e. a null one)?

We draw up a starting-point statement of knowledge of rainforest tree species distribution in New Caledonia by compiling tree occurrences from four distinct sources: (i) herbarium specimens, (ii) plot inventories, (iii) photographs and (iv) other observations collected over several decades. First, we evaluated the geographic and environmental representativeness of this occurrence dataset. Second, we analysed how tree species richness found on the main island (i.e. the γ-diversity) is distributed along altitudinal and rainfall gradients on the two main substrate types. Last, we examined whether the spatial distribution of rainforest tree species is more driven by the environment than by dispersal ability. If species exhibit environmental specialisation, their distribution was hypothesized to be mainly driven by deterministic processes. Furthermore, if species exhibit aggregative distributional patterns, we then concluded that their distribution is mainly shaped by stochastic processes and controlled by dispersal ability.

## Methods

### Study site

New Caledonia is located slightly north of the Tropic of Capricorn (20–23°S, 164–167°E), ∼1500 km east of Australia and 2000 km north of New Zealand. The main island ‘*Grande Terre*’, is long (400 km), narrow (40 km) and accounts for nearly 85 % of the archipelago area. It lies roughly SE : NW and is crossed by a central mountain chain, where the highest peaks reach 1628 m in the north (Mont Panié) and 1618 m in the south (Mont Humboldt).

Most of the UM substrates are located in the southeast of *Grande Terre* in a main massif called the *Grand Massif du Sud* while 12 other smaller UM massifs are scattered along the northwest coast (Fritsch 2012). The UM substrates provide a variety of soils with somewhat unusual characteristics—always a deficiency of phosphorus, potassium and calcium; frequently a high concentration of magnesium; often a low water retention and potentially phytotoxic levels of some metals including nickel, manganese, chromium and cobalt. Obviously, plants must be able to tolerate these soil conditions if they are to establish here (Jaffré 1980). Overall, New Caledonian trees can be classified into three balanced edaphic groupings: UM specialists, non-ultramafic (non-UM) specialists (on volcano-sedimentary or acidic substrates) and substrate-generalists (Ibanez *et al*. 2014).

Rainfalls range widely from 0.6 to 4.5 m year^−1^ and are generally lower on the leeward lowlands of the west coast and higher on the windward mountain slopes of the east-coast due to oro-topography and the eastern trade winds (Météo-France 2007; Terry *et al*. 2008). A combination of rainfall and elevation is commonly used to classify the vegetation into the following: rainforest, dry sclerophyll forest, scrubland called ‘maquis’, savannah, secondary thickets and mangroves (Jaffré 1993; Jaffré *et al*. 2012). Rainforests are the richest vegetation type (more than 2000 native vascular species) and covers ∼3800 km^2^, with 1800 km^2^ on non-UM, 1100 km^2^ on UM and 900 km^2^ on calcareous substrates, mainly located in the *Loyalty Islands*.

### Selection of tree species

We focussed on the distribution of 702 woody tree species (i.e. excluding lianas, tree ferns and palms) reaching a diameter at breast height (DBH at 1.3 m) of 10 cm, at least once in our set of 37 597 trees in the New Caledonian Plant Inventory and Permanent Plot Network (NC-PIPPN). Intraspecific ranks (subspecies and varieties) were merged at the specific rank to ensure uniformity of identification across datasets. The nomenclature of tree species followed the New Caledonian taxonomic name reference Florical (Morat *et al*. 2012).

The NC-PIPPN inventory covers a surface area of ∼15 ha and comprises: (i) 220 plots of 0.04 ha (20 × 20 m), with 30 221 inventoried trees (DBH >5 cm) located across rainforests of *Grande Terre* on both UM (111 plots) and non-UM substrates (89 plots) along a wide range of elevations (5–1292 m on UM and 105–1187 m on non-UM) and rainfalls (1.6–3.5 m year^−1^ on UM and 1.8–3.4 m year^−1^ on non-UM) (see Ibanez *et al*. 2014) and (ii) six recently established 1 ha plots (100 × 100 m), with 7376 inventoried trees (DBH >10 cm) located in rainforests of the Northern Province on non-UM substrates at mid-elevations (240–780 m) and mid-rainfalls (1.3–3.0 m year^−1^).

### Compilation of tree occurrences

Occurrences of the 702 tree species were compiled from four datasets: (i) the NC-PIPPN inventory (29 409 occurrences), (ii) herbarium specimens (22 715 specimens) compiled from the database of the herbarium of the IRD Centre of Noumea (NOU, http://herbier-noumea.plantnet-project.org), (iii) other observations (44 227 observations) from different unpublished inventories used for assessing the flora of areas under consideration for mining exploration or for conservation measures (**[see Supporting Information]**) and (iv) photographs acquired in the field (4326 photographs).

We then checked datasets and removed inaccurate geolocations. We only retained herbarium specimens that were: (i) georeferenced with a Global Positioning System (GPS), (ii) collected by Hugh S. MacKee (the principal contributor to the NOU Herbarium with ∼45 000 specimens) and estimated to have a horizontal accuracy of <500 m according to his online gazetteer (http://phanero.novcal.free.fr) or (iii) labelled with an elevation matching with a difference of ±50 m the elevation extracted from a digital elevation model (DEM).

From the 100 677 initial occurrences, we compiled a dataset of 38 936 (∼40 %) unique occurrences combining species with accurate geolocation: 11 845 from herbarium, 7420 from plots, 18 390 observations and 1281 photographs, used thereafter to describe the known distribution of tree species in New Caledonia. A total of 15 285 occurrences (i.e. <40 % of the whole dataset) were found in rainforests while the remainder occurred in other vegetation types.

### Environmental features

Substrate types (i.e. UM and non-UM) were extracted from a UM substrate map downloaded from the Geographic Portal of New Caledonia (http://www.georep.nc/, DIMENC/SGNC-BRGM 2010). Elevation was extracted from a 50-m resolution digital elevation model (DEM-DTSI 2012) and rainfall from an interpolation model with a resolution of 1 km using mean annual rainfall compiled from 1990 to 2010 (AURELHY model, METEO-FRANCE). Finally, vegetation types were extracted from the vegetation map of the Atlas of New Caledonia (Bonvallot *et al*. 2012). This vegetation map is a broad-scale digitalization (scale of 1/1 600 000) based on aerial photographs in which only the largest rainforest units covering ∼3276 km^2^ were delineated (Jaffré *et al*. 2012).

### Statistical analysis

#### Tree occurrences dataset

First, we subdivided the main island into 5546 cells of 1 min^2^ resolution (1.852 × 1.852 km) and computed in each cell the number of occurrences (i.e. the occurrence density) and the number of species (i.e. the species richness or α-diversity). We then analysed the relative contributions of the four datasets and examined the occurrence and species patterns they produced.

#### Geographical and environmental distributions of γ-diversity

We then focussed on occurrences found exclusively in rainforests, and analysed their distribution as a function of environmental features including (i) the substrate type (UM or non-UM), (ii) elevation and (iii) mean annual rainfall. For each substrate, elevation and rainfall class (bands of 100 m and 0.25 m year^−1^, respectively), we computed the number of occurrences found in rainforests, the observed γ-diversity and the theoretical γ-diversity (calculated by considering that a species occurs between the minimal and maximal class where it had been recorded). We then compared the class distribution of available rainforest area, occurrences, observed and theoretical γ-diversity using linear log–log models of correlation. Finally, we computed species rarefaction curves and Fisher's α diversity index at low, mid and high classes of elevation (<400, 400–800 and >800 m) and rainfall (<2.5, 2.5–3.0 and >3.0 m year^−1^) to avoid sampling bias (see McCain and Grytnes 2010). Occurrence-based rarefaction curves with 95 % confidence intervals were computed using 1000 random permutations (e.g. Gotelli and Colwell 2001).

#### Geographical and environmental distributions of species

Finally, we calculated a basic index of aggregation as the ratio between the number of occurrences and the number of grid-cells intercepted by each dataset (i.e. the mean density of occurrences per grid-cell). At the species level, we computed the Morisita index of aggregation (*I*_mor_) in grid-cells as well as in the entire plots dataset (before removing species duplicates) and used the standardized *I*_mst_ which ranges from −1 to 1. Species with *I*_mst_ ≤ −0.5 are equally distributed across grid-cells or plot inventories while species with *I*_mst_ ≥ 0.5 are aggregated in some grid-cells or plots.

Geographic data processing was performed using Quantum GIS 2.6.0-Brighton (Quantum GIS Development Team, 2014) and statistical analyses using R 2.15.2 (R Foundation for Statistical Computing, Vienna, Austria), including the *vegan* package (Oksanen *et al*. 2013).

## Results

### Tree occurrences dataset

The 702 tree species selected belong to 195 accepted genera and 80 families. At the family level, Myrtaceae were the most common, followed in decreasing order by Cunoniaceae, Sapindaceae, Araliaceae, Sapotaceae, Clusiaceae, Rubiaceae, Lauraceae, Primulaceae, Rutaceae and Apocynaceae. Together these represented 50 % of the total number of occurrences **[see Supporting Information]**. At the species level, the 124 species most-represented (92–491 occurrences per species) contributed to 50 % of the total occurrences. In contrast, the 434 species least-represented (1–50 occurrences per species) contributed to <25 % of the total number of occurrences.

**Figure 1.**
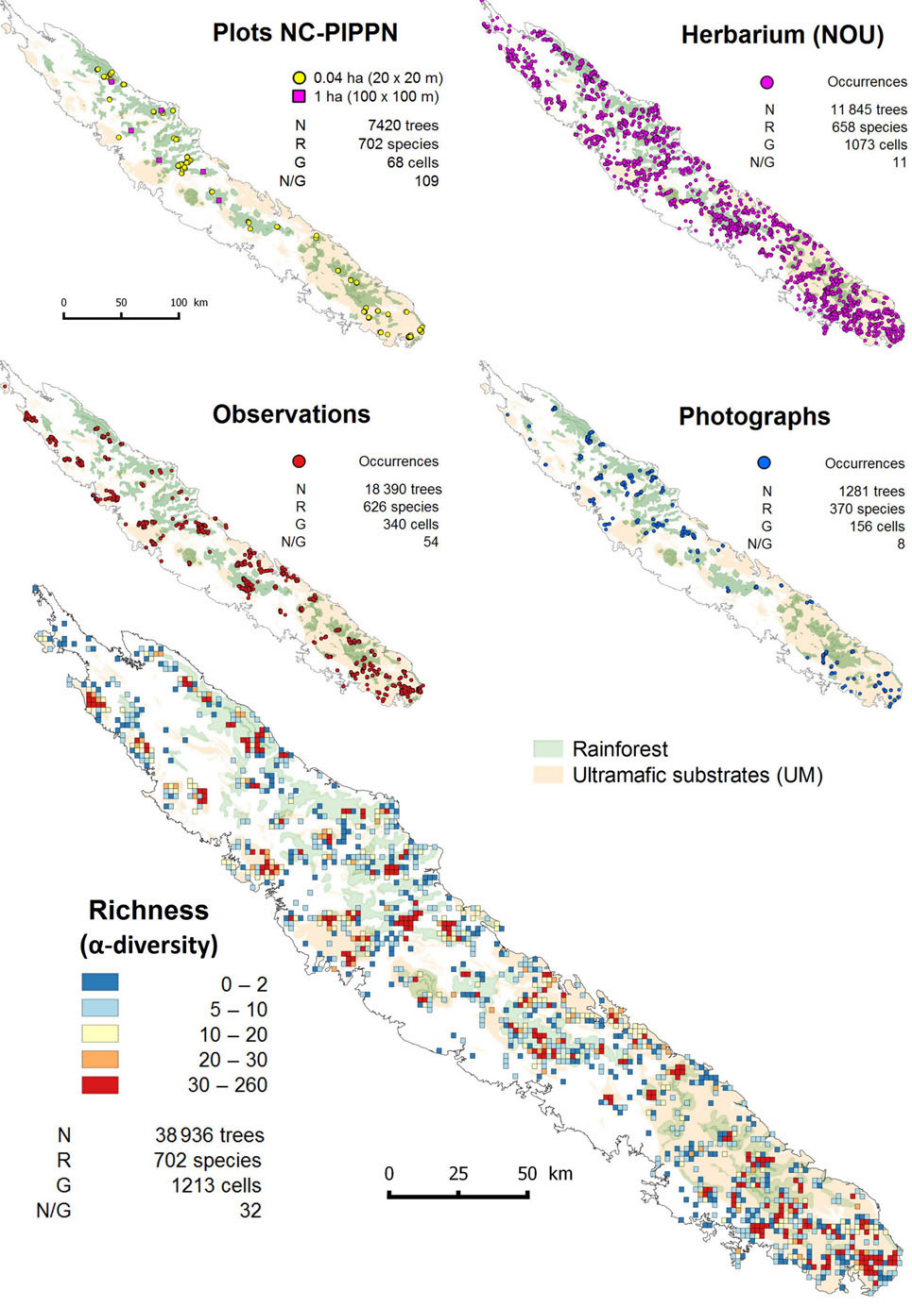
Distribution of the number of tree occurrences (*N*), the number of species or γ-diversity (*R*), number of intercepted cells on a 1 min-resolution grid (*G*) and the occurrences/cells ratio (*N*/*G*) within each dataset and the resulting α-diversity computed for overall occurrences by 1-min cell (1.852 × 1.852 km).

Tree occurrences were distributed into 1213 cells of a regular 1-min grid, which covers ∼22 % of New Caledonia's main island (Fig. 1). Occurrence density ranged from 1 to 1405 per grid-cell (32 on average, ±2 standard error). Occurrences from the plot inventory dataset were the most aggregated data, occurring in only 68 cells with an average of 109 occurrences ±18 per grid-cell. The next most aggregated dataset was of observations (340 grid-cells and 54 occurrences ±5 per grid-cell). This was followed by herbarium specimens (1073 grid-cells and 11 occurrences ±1 per grid-cell) and, last, photographs (156 grid-cells and 8 occurrences ±1 per grid-cell).

Tree occurrences were substantially imbalanced among substrate types (Table 1). More than two-thirds of the occurrences were found on UM substrates while these substrates cover only one-third of the island (Fig. 1). This over-sampling was particularly high in the observation dataset. Thus, the number of occurrences for a given species was strongly correlated with its abundance on UM substrates (Pearson's correlation test *R*^2^ = 0.94, *P*-value <0.001).

**Table 1.** Distribution of land area and tree occurrences in the whole *Grande Terre* (All) and in the outline of rainforests (Forest) for both UM and non-UM substrates. Italic values represent the relative contribution of each classes.

	All			Forest		
	UM	Non-UM	Total	UM	Non-UM	Total
Area (km^2^)	5805	11 469	17 274	1190	2086	3276
	*33.6* *%*	*66.4* *%*		*36.3* *%*	*63.7* *%*	
Occurrences (#)	26 340	12 596	38 936	7009	8276	15 285
	*67.6* *%*	*32.4* *%*		*45.9* *%*	*54.1* *%*	
Herbarium	6626	5219	11 845	2670	3204	5874
	*55.9* *%*	*44.1* *%*		*45.5* *%*	*54.5* *%*	
Plot	3831	3589	7420	780	3074	3854
	*51.6* *%*	*48.4* *%*		*20.2* *%*	*79.8* *%*	
Photograph	498	783	1281	192	599	791
	*38.9* *%*	*61.1* *%*		*24.3* *%*	*75.7* *%*	
Observation	15 385	3005	18 390	3367	1399	4766
	*83.7* *%*	*16.3* *%*		*70.6* *%*	*29.4* *%*	

**Figure 2.**
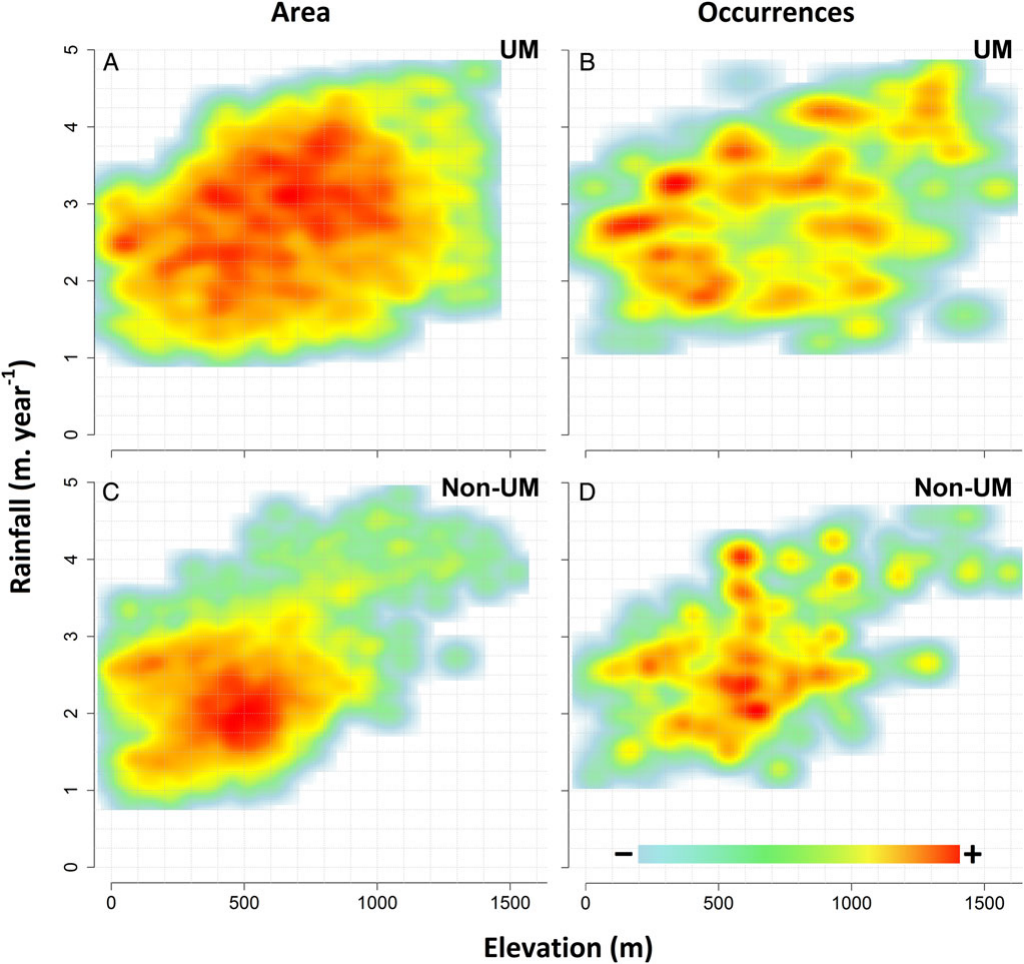
Relative density distributions of land area (A and C) and tree occurrences (B and D) on UM substrate (A and B) and non-UM substrates (C and D) along the rainfall and elevation gradients.

Overall, tree occurrences covered most of the elevation—­rainfall combinations available on both UM and non-UM substrates (Fig. 2). As a result, we observed a significant correlation between the number of occurrences and the available rainforest area along the elevation gradient (*R*^2^ = 0.71 on UM and 0.64 on non-UM, *P*-value <0.001 in both cases). This correlation was weaker along the rainfall gradient (*R*^2^ = 0.45 on UM and 0.34 on non-UM, *P*-value <0.01 and 0.05, respectively, Table 2).

**Table 2.** Linear log–log correlation between the available rainforest area (AREA), the number of occurrences (*N*), the observed γ-diversity (*R*_obs_) and the theoretical γ-diversity (*R*_theo_) along the elevation gradient and the rainfall gradient and on UM and non-UM substrates separately (*P < 0.05, **P < 0.01, ***P < 0.001).

Model	Gradient	Substrates	Slope (SE)	Intercept (SE)	*R*^2^	*F* value	Df	*P*-value
log(*N*) = *a* × log(AREA) + *b*	Elevation	UM	0.44 (0.08)	−1.52 (0.30)	0.71	34.65	14	***
		Non-UM	0.52 (0.10)	−1.39 (0.44)	0.65	25.78	14	***
	Rainfall	UM	0.85 (0.25)	−0.62 (0.86)	0.45	11.32	14	**
		Non-UM	0.87 (0.32)	−0.71 (1.16)	0.34	7.33	14	*
log(R_obs_) = *a* × log(*N*) + *b*	Elevation	UM	0.83 (0.06)	7.56 (0.22)	0.92	163.9	15	***
		Non-UM	0.81 (0.06)	7.56 (0.23)	0.92	175.3	15	***
	Rainfall	UM	0.64 (0.04)	7.03 (0.12)	0.96	334.1	13	***
		Non-UM	0.84 (0.06)	7.56 (0.22)	0.95	229.9	13	***
log(R_obs_) = *a* × log(AREA) + *b*	Elevation	UM	0.36 (0.06)	6.31 (0.22)	0.74	40.27	14	***
		Non-UM	0.44 (0.09)	6.49 (0.36)	0.65	25.88	14	***
	Rainfall	UM	0.49 (0.16)	6.52 (0.55)	0.40	8.84	13	*
		Non-UM	0.83 (0.26)	7.38 (0.94)	0.44	10.16	13	**
log(R_theo_) = *a* × log(AREA) + *b*	Elevation	UM	0.37 (0.06)	−1.01 (0.26)	0.7	32.9	14	***
		Non-UM	0.32 (0.07)	−1.16 (0.28)	0.63	24	14	***
	Rainfall	UM	0.57 (0.18)	−0.60 (0.60)	0.44	10.27	13	**
		Non-UM	0.78 (0.27)	−0.13 (0.96)	0.39	8.44	13	*
log(R_theo_) = *a* × log(*N*) + *b*	Elevation	UM	0.89 (0.06)	0.42 (0.20)	0.93	213.8	15	***
		Non-UM	0.65 (0.07)	−0.23 (0.27)	0.84	82.05	15	***
	Rainfall	UM	0.70 (0.05)	−0.13 (0.17)	0.94	214.3	13	***
		Non-UM	0.80 (0.07)	0.11 (0.31)	0.90	111.1	13	***
log(*R*_obs_) = *a* × log(R_theo_) + *b*	Elevation	UM	0.93 (0.03)	7.18 (0.09)	0.98	734.8	15	***
		Non-UM	1.09 (0.13)	7.45 (0.34)	0.83	72.02	15	***
	Rainfall	UM	0.89 (0.04)	7.10 (0.10)	0.97	486.1	13	***
		Non-UM	1.00 (0.05)	7.33 (0.14)	0.97	480.3	13	***

However, Fig. 2 reveals that occurrences on UM substrates were particularly under-represented at mid-elevations (500–800 m) and for mid-rainfalls (2.3–3.0 m year^−1^), while they were over-represented at high elevations (above 1200 m) and for rainfalls (above 4.0 m year^−1^). On non-UM substrates, occurrences were concentrated in a narrow elevation range (500–700 m) and also over-represented at high elevations (above 800 m) and for rainfalls (above 3.0 m year^−1^).

**Figure 3.**
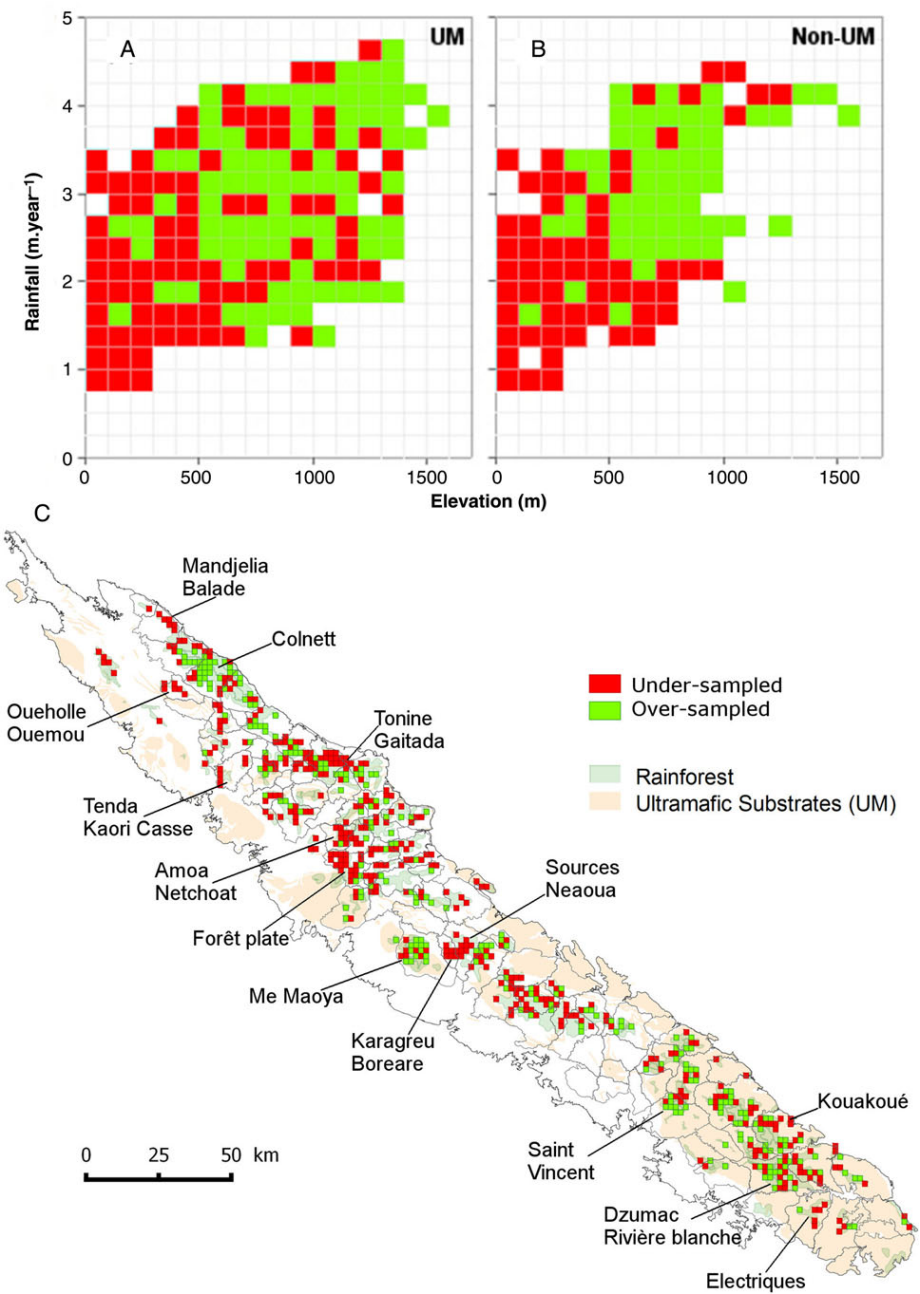
Environmental representativeness of occurrences on UM (A) and non-UM substrates (B) and geographical projection of no-data cells (C). Cells in red and green do not contain data. Cells in red are under-represented in our dataset and so are priorities for future botanical surveys to improve the knowledge of species distribution.

Figure 3A and B shows under- and over-represented regions of the environmental space. On both substrates, below 500 m elevation and 2 m year^−1^ rainfall, occurrences were under-represented with regard to the relative rainforest surfaces. When projected on a map, under-represented areas covered 1873 km^2^ of rainforests (i.e. 57 % of the total rainforest area) including 975 km^2^ (52 %) on UM substrates (Fig. 3C). The number of occurrences in under-represented areas was null in two-thirds of the grid-cells (no-data cells) and ranged from 1 to 1405 in the other third. Among major orographic massifs with no-data cells, those presenting an over-represented environment include ‘Colnett’, ‘Me Maoya’ or ‘Saint Vincent’ while those with an under-represented environment include ‘Mandjelia/Balade’, ‘Tonine/Gaitada’ ‘Forêt plate’, ‘Source Neaoua’, ‘Karagreu/Boreare’ and ‘Kouakoué’.

### Geographical and environmental distributions of γ-diversity

**Figure 4.**
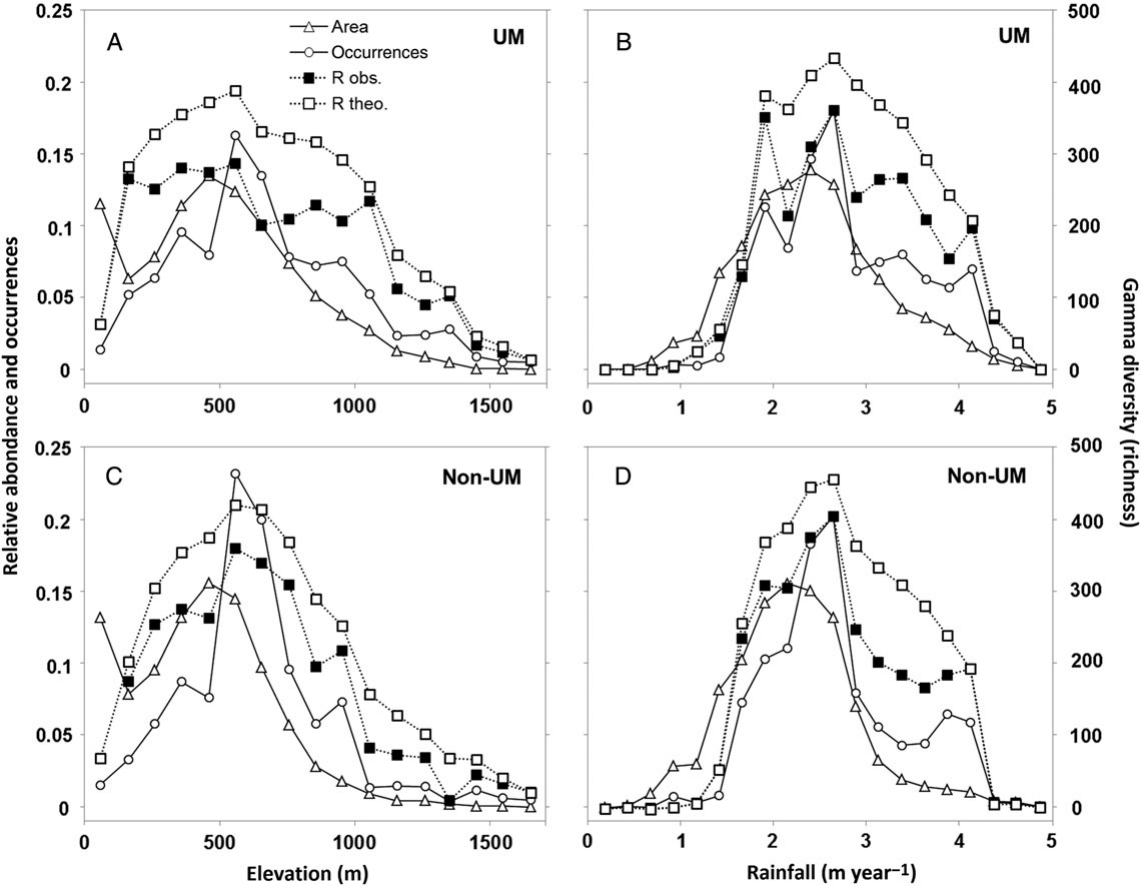
Distribution of observed and theoretical γ-diversities, the density of rainforest area and the density of tree occurrences on UM substrates (A and B) and non-UM substrates (C and D) along the elevation and rainfall gradients.

Overall, the α-diversity (i.e. the total number of species per 1-min cell) was strongly correlated with the total number of occurrences (Pearson's correlation test, *R*^2^ = 0.86, *P*-value <0.001). The observed γ-diversity was strongly log-correlated with the number of occurrences, regardless of the substrate type or the environmental gradient (*R*^2^ > 0.90 and *P*-value <0.001 in all cases, Table 2). Furthermore, the observed γ-diversity was also strongly log-correlated with the theoretical γ-diversity (*R*^2^ > 0.97, *P*-value <0.001) except along the elevation gradient on non-UM substrates, where the correlation was weaker (*R*^2^ = 0.82, *P*-value <0.001, Table 2). Nevertheless we observed important differences between observed and theoretical γ-diversity at mid-elevation and rainfall (Fig. 4). We note that along the elevation gradient, the highest γ-diversity on UM substrates was recorded at low elevation despite a low density of occurrences. Conversely, at high elevations and high rainfalls, occurrences were dense, so the γ-diversity observed deviated from the available area.

**Figure 5.**
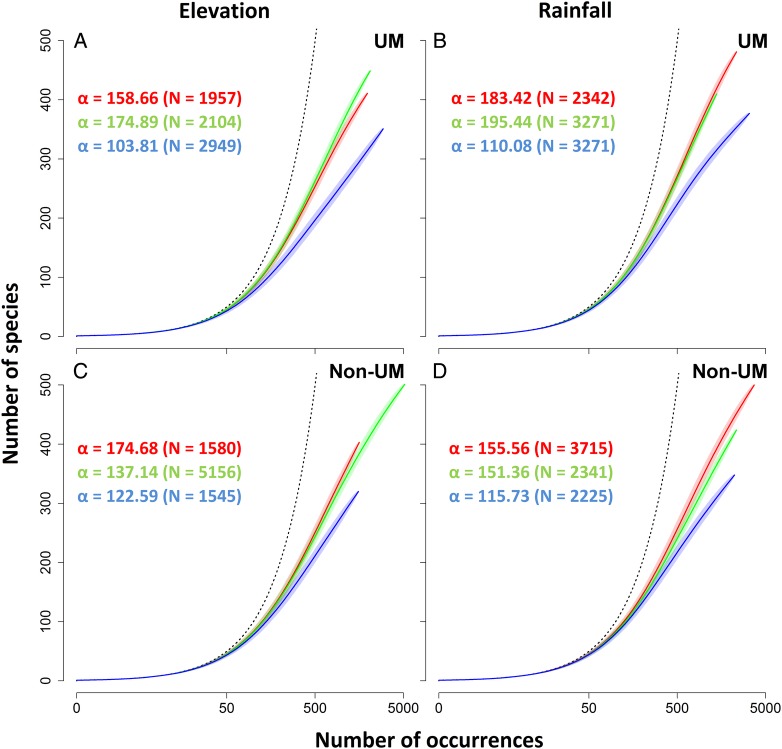
Species richness occurrences-based rarefaction curves compiled for low (red), mid (green) and high (blue) classes of elevation (≤400, 400–800 and >800 m, respectively) and rainfall (≤2.5, 2.5–3.0 and >3.0 m year^−1^, respectively) on UM (A and B) and non-UM substrates (C and D), where α is Fisher's index and *N* is the total number of occurrences (the dotted line is the theoretical maximum rate of accumulation).

However occurrences-based rarefaction curves clearly attest a lower rate of species accumulation at higher classes of elevation and rainfall on both substrates (Fig. 5). Such a decrease was more pronounced on UM than on non-UM substrates. Indeed, on UM substrates, Fisher's *α* decreased by ∼40 % from mid to high elevation or rainfall, while on non-UM substrates it decreased by 25 and 30 % from low to high elevation and rainfall.

### Geographical and environmental distributions of species

Although our list of tree species was drawn from occurrences in rainforest plots, tree species distribution extended to many other vegetation types (see Fig. 1). Indeed, ∼5 % of them (38 species) occurred only in rainforests. Less than 10% (66 species) occurred strictly on UM substrates and 13% (90 species) on non-UM. However, if we consider that a threshold of 90% of the occurrences reveals a species affiliation to a substrate, then 29% (205 species) were affiliated to UM and 23% (161 species) to non-UM. Finally ∼80 % of the rainforest species (561 species) occurred once in ‘*maquis*’ through a total number of 18 503 occurrences.

With respect to other environmental features, half of the species exhibited an elevation tolerance (the difference between the minimum and maximum elevation where a species has been recorded) higher than 895 m (891 ± 332 m on average) and a rainfall tolerance higher than 2.4 m year^−1^ (2.2 ± 0.8 m year^−1^ on average), with no significant deviation according to affiliations to substrate (Kruskal–Wallis rank sum test, *P*-values >0.05). This range of tolerances represents more than half of the elevation and rainfall ranges available across the rainforest of *Grande Terre.* In contrast, the vast majority of species exhibited a spatial distribution highly aggregated at the scale of the *Grande Terre* (92 % of the species with *I*_mst_ ≥ 0.5) as well as in plots (90 % of the species with *I*_mst_ ≥ 0.5).

## Discussion

### Tree occurrences dataset

We compiled occurrences from herbarium specimens, plot inventories, photographs and observations to draw up a first assessment of tree distribution and diversity in New Caledonian rainforests. These occurrences cover almost a quarter of the main island, *Grande Terre*, providing a fairly comprehensive view of the actual distribution of rainforest tree species. Herbarium specimens have long been used to study species and diversity distributions (Lavoie 2013) and the biases associated with a taxonomic approach are widely recognized (Ahrends *et al*. 2011). Species accumulation is more rapid when using herbarium specimens than when using plot inventories but their relative abundance is not reliable (e.g. Garcillán and Ezcurra 2011). To our knowledge, the use of field photographs is less common. The development of GPS and photographic technologies now generates a huge amount of high-quality georeferenced and retrospectively verifiable information. In our study, even though this data source was less substantial, we stress that in the future it is likely to become a critical source of reliable data, by involving parataxonomists, in particular, through collaborative networks (e.g. Basset *et al*. 2004). Even if observations may be more doubtful than from other datasets (i.e. there is no way to check data *a posteriori*), these rapid botanical surveys provide the very substantial quantity of data required to enhance our understanding of species distribution (Guitet *et al*. 2014). The complementarity of these datasets provides information on both the spatial and the ecological distribution of taxa and an assessment of γ-diversity.

The dataset is unbalanced with regard to the number of occurrences found on UM substrates (two-thirds of the dataset). This pattern results from a huge dataset collected over decades on UM substrates to assess and anticipate the environmental impacts of mining activities (McCoy *et al*. 1999). In a cruel irony, our knowledge of tree species distribution on UM has increased in direct proportion to the decline in rainforest areas. In addition, the mismatch between the number of occurrences and available rainforest areas along the elevation and rainfall gradients reveals substantially unknown rainforest areas (notably at low elevation and with rainfall). These areas (see Fig. 3) should be sampled in priority to improve the representativeness of our occurrence data and subsequently to enhance our knowledge of New Caledonian tree species distribution. In the face of the high level of threat to New Caledonian rainforests, this information is critical to build reliable species distribution models under the current climate and also under putative future climatic conditions.

### Geographical and environmental distributions of γ-diversity

The log–log correlations between observed or theoretical species γ-diversity and the available land area along the elevation gradient suggest that elevation impacts γ-diversity mainly through the so-called ‘area effect’ as a consequence of the basic species/area relationship (Sanders 2002). This effect was also found to drive plant γ-diversity on other high-elevation islands, including the vascular flora of Borneo (Grytnes *et al*. 2008), the palm flora of New Guinea (Bachman *et al*. 2004), the epiphytic flora of Taiwan (Hsu *et al*. 2014) and the fern flora of La Réunion Island (Karger *et al*. 2011). At the highest elevations and rainfalls, the species accumulation rates (α-Fisher) reveal a faster saturation of species richness. This may reflect a bias in our species selection method focussed on plots rarely distributed in such extremes values. However, few tree species are known to be high-elevation specialists in New Caledonia (Nasi *et al*. 2002). Furthermore, elevation in New Caledonia remains too low to record abiotic factors such as extreme low temperatures, freezing or extreme levels of solar radiation which can radically change a flora due to plant physiological limitations (Ghalambor *et al*. 2006; McCain and Grytnes 2010). Finally, the weaker correlation between γ-diversity and area along the rainfall gradient suggests that rainfall is likely to act as a stronger environmental driver of γ-diversity than elevation.

### Geographical and environmental distributions of species

Most of the tree species selected from rainforest plots occurred beyond the rainforest boundaries (∼60 %). This habitat transgression may be explained by a high tolerance of some species to open habitats such as ‘maquis’. However, it could also be partially due to the scale of the digitalization of the vegetation map, likely to be inappropriate for exhaustively delineating rainforests which are highly fragmented from low to mid-elevation (Morat *et al*. 1999). This fragmentation results from a dramatic decrease in rainforest areas, which now occur on only 50 % of the pre-human surfaces (Jaffré *et al*. 1998). As a consequence, occurrences recorded in small relict patches were not included in the rainforest dataset but they did contribute in identifying the range of species tolerance with respect to substrate, elevation and rainfall.

Surprisingly, few species are substrate-specialists and our findings depart markedly from a balanced distribution in the three edaphic compartments (UM, non-UM and generalist). The adjective ‘affiliated’ should be used rather than ‘specialist’ since some edaphic transgressions could be explained by soil properties that could mitigate deficiencies and toxicity arising on UM substrates rather than by the substrate itself (Read *et al*. 2006; Ibanez *et al*. 2014). This tolerance to such contrasting habitats leads to an imbalance in species frequency; few were very abundant (124 species that account for 50 % of our dataset) while the vast majority remained rare in our dataset. Hyperdominance or oligarchy has previously been reported in other locations (Pitman *et al*. 2001, 2013; ter Steege *et al*. 2013), including in Pacific islands (Keppel *et al*. 2011), and is commonly related to low environmental heterogeneity, combined with a tendency towards species–habitat associations rather than obligate associations. In New Caledonia, tree oligarchic species always occur beyond the boundaries of substrates and rainforest habitat, even though they are clearly affiliated with UM substrate and rainforest. Thus, while constraints provided by UM substrates are often invoked to explain the originality and the diversification of the New Caledonian flora (Jaffré *et al*. 1987; Pintaud and Jaffré 2001; Pillon *et al*. 2010; Barrabé 2013), our findings nuance the specific relationship between UM substrate and trees distribution. They suggest that the large spatial variability in environmental conditions in UM rainforests could also have been involved in the diversification processes.

Finally, the large tolerance of species to UM substrates, elevation and rainfall contrasts with their spatial aggregation at the scale of *Grande Terre* as well as within plots network. This pattern supports the hypothesized low dispersibility among rainforest tree species (Seidler and Plotkin 2006). The loss of dispersibility in island plants is one of the ‘insular syndromes’ described by Carlquist (Carlquist 1974). Three hypotheses have been proposed by Carlquist to explain the loss of dispersibility in island plants: (i) the ‘precinctiveness’, which means that most seedlings germinate close to the parent plant because these habitats are more likely to be favourable than those further away; (ii) some island plants have shifted from a pioneering plant syndrome (*r*-strategists) to an exacerbated forest syndrome (*K*-strategists) and (iii) contact with the original dispersal vector may have been lost. The first hypothesis fits well with the observed aggregation of rainforest tree species while the third could be consistent with the low diversity of potential animal dispersal agents (Carpenter *et al*. 2003). However the second hypothesis runs counter to the very high tolerance of species to environmental gradients, including the observed transgressions beyond the habitat boundaries.

As a result, although the γ-diversity follows classical variations (e.g. hump-shaped) along environmental gradients, the distribution of individual species results more from stochastic processes rather than from deterministic ones (Leibold 1995; Chase and Myers 2011; Rosindell *et al*. 2011). This is in line with previous results from Ibanez *et al*. (2014) showing that the α-diversity is particularly low and the β-diversity particularly high in New Caledonian rainforests.

## Conclusions

This study is the first to attempt to describe tree diversity in New Caledonian rainforests through extensive datasets collected over several decades. The collection effort is clearly critical if a realistic view of the observed γ-diversity is to be obtained, while the complementarity of several data collection methods provides both a comprehensive and dense coverage of tree species distribution. The wide ranges of tree species distribution with respect to substrates, elevation and rainfall contrast (i) with their spatial aggregation, (ii) with the small extent of rainforest core and (iii) with the lowest γ-diversity observed at high elevation or rainfall. Our results suggest a uniform γ-diversity from low to mid-elevations (<800 m) and rainfalls (<2.5 m year^−1^), mostly dependent of occurrences and availability of rainforest areas. Above these thresholds, the decrease in the γ-diversity could be more related to the increase in rainfall through biological processes that we must further investigate in the future. Lastly, this study calls for new botanical data to be collected, mainly in non-UM rainforests under low elevation and low rainfall, to better estimate the total γ-diversity with respect to the large amplitude of ecological conditions available on *Grande Terre*.

## Sources of Funding

Our work was partially funded by the DDEE-SIEC, ‘La direction du développement économique et de l’environnement-Service impact environmental et conservation’ of the Northern Province of New Caledonia.

## Contributions by the Authors

P.B. conceived the study, managed the database, compiled the datasets, proceeded with the GIS-analysis and led the writing of this article. T.I. conceived the study, led the statistical analyses and actively contributed to the writing. R.P. actively contributed to the data compilation, the statistical analyses and the writing. H.V. was involved in the collection of many tree species occurrences in all the datasets used in this article. V.H. was also involved in the collection of many tree species occurrences in all the datasets used in this article. E.B. was fully involved in the setting up of 1 ha plots and reviewed the final manuscript. T.J. contributed to the overall coherence, robustness and balance of this article by providing the essential references and contributing from his experience of the ecology of New Caledonian forests, especially those on UM soils.

## Conflict of Interest Statement

None declared.

## Supporting Information

The following additional information is available in the online version of this article –

**File S1.** List of the 702 tree species according to their endemic (*E*) or indigenous (*I*) status, the total number of occurrences (*N*), the number of occurrences on ultramafic (UM) substrates, the number of occurrences in rainforest (Forest) and the ranges of elevation and rainfall calculated by the difference between the maximum and the minimum values where the species have been recorded.

**File S2.** Simplified list of reports related to inventory studies and surveys in New Caledonian forests that have most contributed to the ‘observations’ tree occurrences dataset.

Additional Information
