# Supplementary material for: Environmental correlates for tree occurrences, species distribution and richness on a high-elevation tropical island
Source: AoB Plants. 2015 Jul 10;7:plv075. doi: 10.1093/aobpla/plv075 (PMC4561634; doi:10.1093/aobpla/plv075)
Supplement: Additional Information [file supp_7_plv075_index.html]

Environmental correlates for tree occurrences, species distribution and richness on a high-elevation tropical island — Additional Information 

# Environmental correlates for tree occurrences, species distribution and richness on a high-elevation tropical island

## Additional Information

Additional Information

- Supplementary File 1 - doc file
- Supplementary File 2 - doc file
