# Supplementary material for: Environmental correlates for tree occurrences, species distribution and richness on a high-elevation tropical island
Source: AoB Plants. 2015 Jul 10;7:plv075. doi: 10.1093/aobpla/plv075 (PMC4561634; doi:10.1093/aobpla/plv075)
Supplement: Additional Information [file supp_plv075_plv075supp_file1.doc]

| Family | Taxa | Statut | N | UM | Forest | Rainfall  range (m.y-1) | Elevation  range (m) |
| --- | --- | --- | --- | --- | --- | --- | --- |
| Alseuosmiaceae | Periomphale balansae Baill. | E | 98 | 64 | 65 | 3.0 | 1451 |
| Amborellaceae | Amborella trichopoda Baill. | E | 85 | 4 | 72 | 2.4 | 1169 |
| Anacardiaceae | Euroschinus aoupiniensis M.Hoff | E | 10 | 0 | 9 | 0.7 | 636 |
| Anacardiaceae | Euroschinus elegans Engl. | E | 71 | 71 | 16 | 2.4 | 963 |
| Anacardiaceae | Euroschinus rubromarginatus Baker f. | E | 53 | 52 | 14 | 2.9 | 1043 |
| Anacardiaceae | Euroschinus vieillardii Engl. | E | 35 | 2 | 18 | 1.8 | 848 |
| Anacardiaceae | Semecarpus atra (G.Forst.) Vieill. | E | 18 | 6 | 9 | 2.2 | 680 |
| Anacardiaceae | Semecarpus neocaledonica Engl. | E | 96 | 93 | 18 | 1.9 | 922 |
| Annonaceae | Meiogyne baillonii (Guillaumin) Heusden | E | 44 | 31 | 16 | 2.9 | 781 |
| Annonaceae | Meiogyne lecardii (Guillaumin) Heusden | E | 15 | 3 | 12 | 0.9 | 539 |
| Annonaceae | Meiogyne tiebaghiensis (Däniker) Heusden | E | 106 | 83 | 28 | 2.9 | 945 |
| Annonaceae | Xylopia dibaccata Däniker | E | 12 | 8 | 1 | 1.8 | 163 |
| Annonaceae | Xylopia vieillardii Baill. | E | 86 | 38 | 37 | 2.2 | 1100 |
| Annonaceae | Goniothalamus sp. "Vandrot 296" | E | 18 | 0 | 17 | 2.3 | 428 |
| Annonaceae | Goniothalamus obtusatus (Baill.) R. M. K. Saunders | E | 28 | 6 | 16 | 2.9 | 596 |
| Apiaceae | Apiopetalum velutinum Baill. | E | 41 | 41 | 31 | 2.9 | 1221 |
| Apocynaceae | Alstonia balansae Guillaumin | E | 16 | 9 | 1 | 1.5 | 572 |
| Apocynaceae | Alstonia coriacea Pancher ex S.Moore | E | 17 | 17 | 7 | 2.0 | 896 |
| Apocynaceae | Alstonia lanceolata Van Heurck | E | 13 | 0 | 12 | 1.1 | 390 |
| Apocynaceae | Alstonia lanceolifera S.Moore | E | 9 | 9 | 7 | 1.3 | 829 |
| Apocynaceae | Alstonia lenormandii Van Heurck & Müll.Arg. | E | 117 | 94 | 16 | 2.7 | 1002 |
| Apocynaceae | Alstonia odontophora Boiteau | E | 19 | 19 | 6 | 2.0 | 1028 |
| Apocynaceae | Alstonia sphaerocapitata Boiteau | E | 12 | 2 | 10 | 1.0 | 384 |
| Apocynaceae | Alstonia vieillardii Van Heurck | E | 37 | 36 | 18 | 2.4 | 737 |
| Apocynaceae | Cerberiopsis candelabra Vieill. | E | 76 | 71 | 8 | 2.3 | 547 |
| Apocynaceae | Neisosperma brevituba (Boiteau) Boiteau | E | 14 | 0 | 12 | 2.3 | 554 |
| Apocynaceae | Neisosperma miana (Baill. ex White) Boiteau | E | 92 | 87 | 23 | 2.8 | 764 |
| Apocynaceae | Ochrosia balansae (Guillaumin) Baill. ex Guillaumin | E | 60 | 57 | 9 | 2.2 | 972 |
| Apocynaceae | Ochrosia grandiflora Boiteau | E | 9 | 3 | 7 | 1.1 | 497 |
| Apocynaceae | Ochrosia silvatica Däniker | E | 15 | 12 | 4 | 1.5 | 893 |
| Apocynaceae | Rauvolfia balansae (Baill.) Boiteau | E | 148 | 97 | 66 | 3.0 | 1383 |
| Apocynaceae | Rauvolfia semperflorens (Müll.Arg.) Schltr. | E | 103 | 86 | 13 | 2.7 | 1062 |
| Apocynaceae | Alstonia costata (G.Forst.) R.Br. | A | 41 | 15 | 20 | 2.2 | 681 |
| Apocynaceae | Tabernaemontana cerifera Pancher & Sebert | E | 195 | 166 | 42 | 3.1 | 1177 |
| Aquifoliaceae | Ilex sebertii Pancher & Sebert | E | 267 | 209 | 56 | 3.0 | 1417 |
| Araliaceae | Meryta balansae Baill. | E | 103 | 34 | 75 | 2.6 | 975 |
| Araliaceae | Meryta coriacea Pancher ex Baill. | E | 186 | 179 | 70 | 3.5 | 1538 |
| Araliaceae | Meryta lecardii (R.Vig.) Lowry & F.Tronchet, ined. | E | 47 | 43 | 8 | 2.2 | 823 |
| Araliaceae | Meryta oxylaena Baill. | E | 38 | 9 | 27 | 2.6 | 851 |
| Araliaceae | Meryta pachycarpa Baill. | E | 13 | 13 | 2 | 2.4 | 948 |
| Araliaceae | Meryta pedunculata Lowry & F.Tronchet, ined. | E | 5 | 0 | 5 | 1.0 | 349 |
| Araliaceae | Meryta schizolaena Baill. | E | 3 | 0 | 2 | 1.1 | 419 |
| Araliaceae | Schefflera candelabrum Baill. | E | 16 | 0 | 9 | 1.1 | 690 |
| Araliaceae | Plerandra neocaledonica Lowry, G.M.Plunkett & Frodin | E | 11 | 11 | 6 | 1.2 | 1105 |
| Araliaceae | Schefflera pseudocandelabrum R.Vig. | E | 22 | 5 | 20 | 1.3 | 868 |
| Araliaceae | Polyscias bracteata (R.Vig.) Lowry | E | 127 | 79 | 51 | 3.3 | 1222 |
| Araliaceae | Polyscias dioica (Vieill. ex Pancher) Harms | E | 491 | 445 | 94 | 3.7 | 1422 |
| Araliaceae | Polyscias cissodendron (C.Moore & F.Muell.) Harms | A | 25 | 1 | 16 | 2.5 | 844 |
| Araliaceae | Polyscias lecardii (R.Vig.) Lowry | E | 20 | 2 | 18 | 2.5 | 1039 |
| Araliaceae | Polyscias vieillardii (Baill.) Lowry & G.M.Plunkett | E | 40 | 16 | 18 | 2.5 | 638 |
| Araliaceae | Polyscias otopyrena (Baill.) Lowry & G.M.Plunkett | E | 62 | 54 | 28 | 2.3 | 1139 |
| Araliaceae | Polyscias mackeei Lowry & G.M.Plunkett | E | 40 | 38 | 2 | 1.4 | 569 |
| Araliaceae | Plerandra nono (Baill.) Lowry, G.M.Plunkett & Frodin | E | 23 | 22 | 8 | 1.6 | 704 |
| Araliaceae | Plerandra gordonii Lowry, G.M.Plunkett & Frodin | E | 114 | 113 | 11 | 2.4 | 931 |
| Araliaceae | Plerandra moratiana Lowry & G.M.Plunkett, ined. | E | 2 | 1 | 2 | 0.3 | 258 |
| Araliaceae | Plerandra gabriellae (Baill.) Lowry, G.M.Plunkett & Frodin | E | 149 | 68 | 76 | 3.1 | 1086 |
| Araliaceae | Plerandra plerandroides (R.Vig.) Lowry, G.M.Plunkett & Frodin | E | 40 | 0 | 32 | 2.4 | 1452 |
| Araliaceae | Plerandra crassipes (Baill.) Lowry, G.M.Plunkett & Frodin | E | 56 | 55 | 42 | 3.3 | 1092 |
| Araliaceae | Plerandra veitchii (Carrière) Lowry, G.M.Plunkett & Frodin | E | 10 | 5 | 0 | 2.0 | 508 |
| Araliaceae | Plerandra osyana (Veitch ex Regel) Lowry, G.M.Plunkett & Frodin | E | 64 | 25 | 42 | 2.5 | 1083 |
| Araliaceae | Plerandra leptophylla (Veitch ex T.Moore) Lowry, G.M.Plunkett & Frodin | E | 26 | 0 | 21 | 2.6 | 666 |
| Araliaceae | Plerandra baillonii (R.Vig.) Lowry, G.M.Plunkett & Frodin | E | 1 | 0 | 1 | 0.0 | 0 |
| Araliaceae | Plerandra reginae (Linden ex W.Richards) Lowry, G.M.Plunkett & Frodin | E | 57 | 52 | 38 | 2.5 | 1077 |
| Araliaceae | Plerandra pancheri (Baill.) Lowry, G.M.Plunkett & Frodin | E | 45 | 6 | 28 | 2.8 | 735 |
| Araliaceae | Plerandra veilloniorum Bernardi ex Lowry, G.M.Plunkett & Frodin | E | 7 | 0 | 7 | 1.7 | 378 |
| Araliaceae | Plerandra taomensis Lowry, G.M.Plunkett & Frodin | E | 4 | 4 | 3 | 0.5 | 154 |
| Araucariaceae | Agathis lanceolata Lindl. ex Warb. | E | 109 | 85 | 51 | 2.4 | 1106 |
| Araucariaceae | Agathis montana de Laub. | E | 38 | 0 | 38 | 2.0 | 1007 |
| Araucariaceae | Agathis moorei (Lindl.) Mast. | E | 65 | 24 | 30 | 3.2 | 1027 |
| Araucariaceae | Agathis ovata (C.Moore ex Vieill.) Warb. | E | 84 | 84 | 26 | 2.4 | 1170 |
| Araucariaceae | Araucaria bernieri J.Buchholz | E | 53 | 51 | 7 | 2.6 | 907 |
| Araucariaceae | Araucaria montana Brongn. & Gris | E | 51 | 44 | 16 | 2.6 | 1152 |
| Araucariaceae | Araucaria muelleri (Carrière) Brongn. & Gris | E | 28 | 28 | 1 | 1.2 | 828 |
| Araucariaceae | Araucaria nemorosa de Laub. | E | 10 | 10 | 7 | 0.6 | 267 |
| Asparagaceae | Cordyline neocaledonica (Baker) B.D.Jacks. | E | 28 | 18 | 12 | 2.6 | 1010 |
| Atherospermataceae | Nemuaron vieillardii (Baill.) Baill. | E | 86 | 59 | 54 | 2.6 | 1451 |
| Balanopaceae | Balanops oliviformis Baill. | E | 15 | 0 | 14 | 2.7 | 589 |
| Balanopaceae | Balanops pachyphylla Baill. ex Guillaumin | E | 66 | 57 | 23 | 2.8 | 1084 |
| Balanopaceae | Balanops pancheri Baill. | E | 147 | 129 | 16 | 3.0 | 1043 |
| Balanopaceae | Balanops sparsiflora (Schltr.) Hjelmq. | E | 64 | 45 | 35 | 2.4 | 1451 |
| Balanopaceae | Balanops vieillardii Baill. | E | 48 | 42 | 19 | 1.8 | 1006 |
| Balanopaceae | Balanops sp. "Munzinger 6400" | E | 10 | 0 | 10 | 1.5 | 341 |
| Bignoniaceae | Deplanchea speciosa Vieill. | E | 217 | 197 | 43 | 2.6 | 853 |
| Burseraceae | Canarium oleiferum Baill. | E | 108 | 101 | 17 | 2.6 | 716 |
| Burseraceae | Canarium trifoliolatum Engl. | E | 5 | 4 | 2 | 0.8 | 428 |
| Burseraceae | Canarium whitei Guillaumin | E | 9 | 9 | 2 | 0.7 | 404 |
| Calophyllaceae | Calophyllum caledonicum Vieill. ex Planch. & Triana | E | 305 | 185 | 114 | 3.4 | 946 |
| Calophyllaceae | Calophyllum inophyllum L. | A | 8 | 4 | 1 | 1.0 | 592 |
| Calophyllaceae | Mammea neurophylla (Schltr.) Kosterm. | E | 11 | 4 | 4 | 2.9 | 1437 |
| Cannabaceae | Celtis conferta Planch. | A | 38 | 34 | 1 | 2.3 | 462 |
| Cannabaceae | Celtis hypoleuca Planch. | E | 31 | 30 | 5 | 2.6 | 986 |
| Cannabaceae | Celtis paniculata (Endl.) Planch. | A | 8 | 4 | 1 | 1.0 | 505 |
| Cannabaceae | Trema cannabina Lour. | A | 8 | 3 | 5 | 1.5 | 596 |
| Cardiopteridaceae | Citronella macrocarpa Hürl. | E | 16 | 8 | 8 | 1.7 | 673 |
| Cardiopteridaceae | Citronella sarmentosa (Baill.) Howard | E | 162 | 122 | 81 | 3.0 | 1334 |
| Casuarinaceae | Gymnostoma deplancheanum (Miq.) L.A.S.Johnson | E | 99 | 99 | 2 | 1.6 | 464 |
| Casuarinaceae | Gymnostoma intermedium (J.Poiss.) L.A.S.Johnson | E | 23 | 21 | 8 | 2.4 | 829 |
| Casuarinaceae | Gymnostoma nodiflorum (Thunb.) L.A.S.Johnson | E | 5 | 1 | 0 | 2.4 | 504 |
| Casuarinaceae | Gymnostoma poissonianum (Schltr.) L.A.S.Johnson | E | 113 | 103 | 30 | 3.2 | 1056 |
| Celastraceae | Dicarpellum pancheri (Baill.) A.C.Sm. | E | 139 | 87 | 93 | 3.3 | 1264 |
| Celastraceae | Dicarpellum pronyense (Guillaumin) A.C.Sm. | E | 46 | 43 | 6 | 1.9 | 744 |
| Celastraceae | Maytenus fournieri (Pancher & Sebert) Loes. | E | 172 | 130 | 16 | 3.3 | 1423 |
| Celastraceae | Pleurostylia opposita (Wall.) Alston | A | 10 | 3 | 1 | 0.8 | 412 |
| Celastraceae | Salaciopsis megaphylla (J.Poiss. ex Guillaumin) Loes. | E | 13 | 0 | 13 | 0.9 | 630 |
| Celastraceae | Salaciopsis neocaledonica Baker f. | E | 31 | 13 | 21 | 1.9 | 1145 |
| Celastraceae | Salaciopsis sparsiflora Hürl. | E | 80 | 55 | 31 | 3.0 | 1303 |
| Celastraceae | Elaeodendron cunninghamii Montrouz. | E | 32 | 22 | 7 | 2.1 | 1415 |
| Chloranthaceae | Ascarina rubricaulis Solms | E | 69 | 59 | 46 | 2.9 | 1429 |
| Chloranthaceae | Ascarina solmsiana Schltr. | E | 45 | 10 | 34 | 2.5 | 1436 |
| Chrysobalanaceae | Hunga rhamnoides (Guillaumin) Prance | E | 50 | 32 | 6 | 2.0 | 778 |
| Clusiaceae | Garcinia amplexicaulis Vieill. | E | 151 | 102 | 58 | 3.0 | 1049 |
| Clusiaceae | Garcinia balansae Pierre | E | 272 | 240 | 38 | 2.7 | 1053 |
| Clusiaceae | Garcinia densiflora Pierre | E | 28 | 5 | 23 | 2.3 | 883 |
| Clusiaceae | Garcinia hennecartii Pierre ex Schltr. | E | 122 | 119 | 13 | 2.4 | 1193 |
| Clusiaceae | Garcinia neglecta Vieill. | E | 261 | 214 | 64 | 2.8 | 884 |
| Clusiaceae | Garcinia pedicellata (G.Forst.) Seem. | E | 24 | 13 | 6 | 1.9 | 464 |
| Clusiaceae | Garcinia puat (Montrouz.) Guillaumin | E | 132 | 48 | 67 | 2.8 | 954 |
| Clusiaceae | Garcinia vieillardii Pierre | E | 24 | 0 | 17 | 2.3 | 335 |
| Clusiaceae | Garcinia virgata Vieill. ex Guillaumin | E | 70 | 0 | 43 | 2.7 | 811 |
| Clusiaceae | Montrouziera cauliflora Planch. & Triana | E | 95 | 23 | 75 | 2.7 | 871 |
| Clusiaceae | Montrouziera gabriellae Baill. | E | 58 | 58 | 16 | 2.5 | 928 |
| Clusiaceae | Montrouziera sphaeroidea Pancher ex Planch. & Triana | E | 272 | 247 | 39 | 3.2 | 1060 |
| Clusiaceae | Garcinia sp. "Munzinger 4818" | E | 11 | 0 | 5 | 0.8 | 406 |
| Cornaceae | Alangium villosum (Blume) Wangerin | A | 62 | 42 | 28 | 2.6 | 737 |
| Corynocarpaceae | Corynocarpus dissimilis Hemsl. | E | 39 | 16 | 24 | 3.1 | 1316 |
| Cunoniaceae | Codia discolor (Brongn. & Gris) Guillaumin | E | 251 | 214 | 28 | 3.1 | 1009 |
| Cunoniaceae | Codia incrassata Pamp. | E | 30 | 0 | 21 | 2.5 | 1145 |
| Cunoniaceae | Codia montana J.R.Forst. & G.Forst. | E | 140 | 122 | 15 | 2.4 | 947 |
| Cunoniaceae | Cunonia aoupiniensis Hoogland | E | 15 | 0 | 13 | 2.6 | 1132 |
| Cunoniaceae | Cunonia austrocaledonica Brongn. ex Guillaumin | E | 50 | 7 | 30 | 2.2 | 785 |
| Cunoniaceae | Cunonia balansae Brongn. & Gris | E | 191 | 182 | 49 | 2.9 | 991 |
| Cunoniaceae | Cunonia cerifera Hoogland | E | 18 | 18 | 2 | 1.2 | 391 |
| Cunoniaceae | Cunonia linearisepala (Guillaumin) Bernardi | E | 19 | 15 | 9 | 2.6 | 757 |
| Cunoniaceae | Cunonia montana (Brongn. & Gris) Schltr. | E | 56 | 50 | 44 | 2.9 | 1279 |
| Cunoniaceae | Cunonia pterophylla (Brongn. & Gris) Schltr. | E | 37 | 35 | 17 | 3.0 | 1171 |
| Cunoniaceae | Cunonia pulchella Brongn. & Gris | E | 61 | 17 | 50 | 2.5 | 1251 |
| Cunoniaceae | Cunonia varijuga Hoogland | E | 42 | 37 | 17 | 2.8 | 1215 |
| Cunoniaceae | Geissois hippocastanifolia Guillaumin | E | 10 | 0 | 9 | 1.7 | 897 |
| Cunoniaceae | Geissois hirsuta Brongn. & Gris | E | 31 | 18 | 19 | 1.8 | 856 |
| Cunoniaceae | Geissois montana Vieill. ex Brongn. & Gris | E | 27 | 0 | 19 | 2.9 | 1266 |
| Cunoniaceae | Geissois polyphylla Lécard ex Guillaumin | E | 24 | 0 | 16 | 2.5 | 1143 |
| Cunoniaceae | Geissois pruinosa Brongn. & Gris | E | 117 | 96 | 37 | 2.4 | 1077 |
| Cunoniaceae | Geissois racemosa Labill. | E | 35 | 3 | 20 | 2.6 | 900 |
| Cunoniaceae | Geissois trifoliolata Pancher ex Guillaumin | E | 6 | 2 | 3 | 1.1 | 802 |
| Cunoniaceae | Geissois velutina Guillaumin ex H.C.Hopkins | E | 33 | 32 | 11 | 2.0 | 904 |
| Cunoniaceae | Pancheria billardierei (D.Don) Pamp. | E | 187 | 150 | 25 | 2.8 | 1569 |
| Cunoniaceae | Pancheria brunhesii Pamp. | E | 22 | 5 | 15 | 1.3 | 932 |
| Cunoniaceae | Pancheria calophylla Guillaumin | E | 58 | 54 | 29 | 3.2 | 1165 |
| Cunoniaceae | Pancheria confusa Guillaumin | E | 54 | 54 | 11 | 1.7 | 902 |
| Cunoniaceae | Pancheria gatopensis Vieill. ex Guillaumin | E | 145 | 117 | 23 | 3.2 | 975 |
| Cunoniaceae | Pancheria reticulata Guillaumin | E | 34 | 33 | 8 | 2.9 | 733 |
| Cunoniaceae | Pancheria ternata Brongn. & Gris | E | 118 | 82 | 44 | 3.5 | 1336 |
| Cunoniaceae | Weinmannia dichotoma Brongn. & Gris | E | 66 | 33 | 54 | 3.0 | 1299 |
| Cunoniaceae | Weinmannia paitensis Schltr. | E | 32 | 31 | 28 | 2.6 | 1069 |
| Cunoniaceae | Weinmannia serrata Brongn. & Gris | E | 52 | 3 | 31 | 2.5 | 843 |
| Cunoniaceae | Geissois balansae Brongn. & Gris ex Guillaumin | E | 17 | 1 | 12 | 1.5 | 687 |
| Cunoniaceae | Hooglandia ignambiensis McPherson & Lowry | E | 3 | 0 | 3 | 0.1 | 254 |
| Cunoniaceae | Codia jaffrei H.C.Hopkins & B.Fogliani | E | 39 | 38 | 8 | 1.5 | 554 |
| Cunoniaceae | Spiraeanthemum brongniartianum Schltr. | E | 24 | 1 | 18 | 2.4 | 1351 |
| Cunoniaceae | Spiraeanthemum densiflorum Brongn. & Gris | E | 50 | 3 | 35 | 2.5 | 873 |
| Cunoniaceae | Spiraeanthemum ellipticum Vieill. ex Pamp. | E | 37 | 34 | 35 | 2.9 | 1246 |
| Cunoniaceae | Spiraeanthemum meridionale (Hoogland) Pillon | E | 54 | 51 | 15 | 2.5 | 830 |
| Cunoniaceae | Spiraeanthemum pubescens Pamp. | E | 38 | 24 | 20 | 2.9 | 1490 |
| Dilleniaceae | Hibbertia comptonii Baker f. | E | 8 | 0 | 8 | 1.0 | 253 |
| Dilleniaceae | Hibbertia lucens Brongn. & Gris ex Sebert & Pancher | A | 214 | 190 | 38 | 3.0 | 1272 |
| Dilleniaceae | Hibbertia pancheri (Brongn. & Gris) Briq. | E | 403 | 343 | 68 | 3.3 | 1062 |
| Ebenaceae | Diospyros balansae Guillaumin | E | 4 | 3 | 3 | 1.3 | 125 |
| Ebenaceae | Diospyros brassica F.White | E | 18 | 1 | 17 | 1.3 | 354 |
| Ebenaceae | Diospyros fasciculosa (F.Muell.) F.Muell. | A | 40 | 3 | 12 | 3.3 | 858 |
| Ebenaceae | Diospyros flavocarpa (Vieill. ex P.Parm.) F.White | E | 25 | 1 | 20 | 2.7 | 785 |
| Ebenaceae | Diospyros glans F.White | E | 15 | 14 | 1 | 1.6 | 525 |
| Ebenaceae | Diospyros macrocarpa Hiern | E | 157 | 110 | 88 | 3.6 | 1156 |
| Ebenaceae | Diospyros olen Hiern | A | 252 | 148 | 102 | 3.2 | 1174 |
| Ebenaceae | Diospyros oubatchensis Kosterm. | E | 36 | 0 | 29 | 2.5 | 1200 |
| Ebenaceae | Diospyros parviflora (Schltr.) Bakh.f. | E | 90 | 68 | 21 | 2.1 | 610 |
| Ebenaceae | Diospyros tireliae F.White | E | 3 | 0 | 3 | 0.6 | 216 |
| Ebenaceae | Diospyros trisulca F.White | E | 15 | 1 | 8 | 1.5 | 575 |
| Ebenaceae | Diospyros umbrosa F.White | E | 108 | 98 | 30 | 2.9 | 940 |
| Ebenaceae | Diospyros vieillardii (Hiern) Kosterm. | E | 112 | 99 | 12 | 2.5 | 782 |
| Ebenaceae | Diospyros yaouhensis (Schltr.) Kosterm. | E | 13 | 5 | 5 | 1.7 | 618 |
| Elaeocarpaceae | Elaeocarpus angustifolius Blume | A | 39 | 7 | 24 | 2.6 | 760 |
| Elaeocarpaceae | Elaeocarpus brachypodus Guillaumin | E | 27 | 22 | 24 | 2.2 | 1280 |
| Elaeocarpaceae | Elaeocarpus bullatus Tirel | E | 23 | 2 | 18 | 3.0 | 848 |
| Elaeocarpaceae | Elaeocarpus comptonii Baker f. | E | 10 | 7 | 7 | 1.2 | 348 |
| Elaeocarpaceae | Elaeocarpus dognyensis Guillaumin | E | 84 | 39 | 62 | 2.9 | 1293 |
| Elaeocarpaceae | Elaeocarpus geminiflorus Brongn. & Gris | E | 45 | 1 | 32 | 3.0 | 1264 |
| Elaeocarpaceae | Elaeocarpus guillainii Vieill. | E | 7 | 0 | 5 | 2.0 | 607 |
| Elaeocarpaceae | Elaeocarpus gummatus Guillaumin | E | 18 | 17 | 8 | 2.8 | 910 |
| Elaeocarpaceae | Elaeocarpus hortensis Guillaumin | E | 8 | 4 | 3 | 2.2 | 840 |
| Elaeocarpaceae | Elaeocarpus leratii Schltr. | E | 29 | 28 | 3 | 2.4 | 1008 |
| Elaeocarpaceae | Elaeocarpus speciosus Brongn. & Gris | E | 54 | 44 | 29 | 2.6 | 1336 |
| Elaeocarpaceae | Elaeocarpus vieillardii Brongn. & Gris | E | 7 | 4 | 2 | 1.6 | 419 |
| Elaeocarpaceae | Elaeocarpus weibelianus Tirel | E | 38 | 21 | 16 | 2.8 | 811 |
| Elaeocarpaceae | Elaeocarpus yateensis Guillaumin | E | 116 | 109 | 40 | 2.8 | 1073 |
| Elaeocarpaceae | Sloanea haplopoda (Guillaumin) A.C.Sm. | E | 24 | 22 | 2 | 1.3 | 703 |
| Elaeocarpaceae | Sloanea koghiensis Tirel | E | 20 | 16 | 11 | 1.0 | 842 |
| Elaeocarpaceae | Sloanea lepida Tirel | E | 5 | 1 | 3 | 0.5 | 200 |
| Elaeocarpaceae | Sloanea magnifolia Tirel | E | 25 | 0 | 21 | 2.0 | 608 |
| Elaeocarpaceae | Sloanea montana (Labill.) A.C.Sm. | E | 43 | 22 | 20 | 2.8 | 976 |
| Elaeocarpaceae | Sloanea ramiflora Tirel | E | 22 | 7 | 17 | 1.5 | 529 |
| Elaeocarpaceae | Sloanea raynaliana Tirel | E | 15 | 0 | 13 | 1.0 | 472 |
| Elaeocarpaceae | Elaeocarpus tremulus Tirel & McPherson | E | 12 | 12 | 10 | 0.6 | 612 |
| Elaeocarpaceae | Elaeocarpus coumbouiensis Guillaumin | E | 19 | 18 | 19 | 1.8 | 663 |
| Ericaceae | Styphelia balansae Virot | E | 36 | 24 | 11 | 2.6 | 1360 |
| Ericaceae | Styphelia cymbulae (Labill.) Spreng. | A | 468 | 400 | 72 | 3.6 | 1378 |
| Ericaceae | Styphelia pancheri (Brongn. & Gris) F.Muell. | E | 49 | 47 | 15 | 3.0 | 936 |
| Erythroxylaceae | Erythroxylum novocaledonicum O.E.Schulz | E | 50 | 45 | 2 | 2.7 | 852 |
| Escalloniaceae | Polyosma leratii Guillaumin | E | 22 | 1 | 18 | 2.7 | 702 |
| Escalloniaceae | Polyosma pancheriana Baill. | E | 32 | 31 | 32 | 2.3 | 1350 |
| Euphorbiaceae | Baloghia alternifolia Baill. | E | 35 | 30 | 2 | 2.0 | 575 |
| Euphorbiaceae | Baloghia bureavii (Baill.) Schltr. | E | 32 | 31 | 9 | 1.7 | 1115 |
| Euphorbiaceae | Baloghia inophylla (G.Forst.) P.S.Green | A | 27 | 9 | 15 | 2.2 | 586 |
| Euphorbiaceae | Bocquillonia grandidens Baill. | E | 7 | 0 | 3 | 1.0 | 593 |
| Euphorbiaceae | Bocquillonia lucidula AiryShaw | E | 22 | 3 | 18 | 1.1 | 861 |
| Euphorbiaceae | Bocquillonia nervosa AiryShaw | E | 15 | 0 | 6 | 1.2 | 673 |
| Euphorbiaceae | Bocquillonia phenacostigma AiryShaw | E | 9 | 1 | 7 | 2.7 | 686 |
| Euphorbiaceae | Claoxylon insulanum Müll.Arg. | E | 13 | 1 | 5 | 1.2 | 678 |
| Euphorbiaceae | Cleidion spathulatum Baill. | E | 34 | 1 | 23 | 2.9 | 979 |
| Euphorbiaceae | Cleidion vieillardii Baill. | E | 121 | 93 | 33 | 2.7 | 906 |
| Euphorbiaceae | Codiaeum peltatum (Labill.) P.S.Green | E | 59 | 10 | 25 | 2.9 | 1177 |
| Euphorbiaceae | Macaranga alchorneoides Pax & Liegelsh. | E | 68 | 54 | 27 | 2.2 | 765 |
| Euphorbiaceae | Macaranga corymbosa (Müll.Arg.) Müll.Arg. | E | 45 | 4 | 27 | 2.4 | 1126 |
| Euphorbiaceae | Neoguillauminia cleopatra (Baill.) Croizat | E | 74 | 73 | 13 | 3.1 | 934 |
| Euphorbiaceae | Homalanthus repandus Schltr. | E | 13 | 3 | 9 | 1.7 | 848 |
| Fabaceae | Archidendropsis fournieri (Vieill.) I.C.Nielsen | E | 14 | 2 | 7 | 1.7 | 615 |
| Fabaceae | Archidendropsis fulgens (Labill.) I.C.Nielsen | E | 35 | 7 | 13 | 2.7 | 510 |
| Fabaceae | Archidendropsis glandulosa (Guillaumin) I.C.Nielsen | E | 17 | 10 | 7 | 2.8 | 1003 |
| Fabaceae | Archidendropsis granulosa (Labill.) I.C.Nielsen | E | 144 | 135 | 23 | 2.9 | 800 |
| Fabaceae | Archidendropsis streptocarpa (E.Fourn.) I.C.Nielsen | E | 50 | 8 | 32 | 2.5 | 622 |
| Fabaceae | Arthroclianthus maximus Schindl. | E | 9 | 0 | 5 | 0.9 | 572 |
| Fabaceae | Intsia bijuga (Colebr.) Kuntze | A | 7 | 2 | 1 | 1.2 | 418 |
| Fabaceae | Serianthes sachetae Fosberg | E | 8 | 3 | 3 | 1.3 | 239 |
| Fabaceae | Storckiella pancheri Baill. | E | 108 | 89 | 12 | 2.8 | 1025 |
| Gentianaceae | Fagraea berteroana A.Gray ex Benth. | A | 80 | 34 | 29 | 2.4 | 953 |
| Gesneriaceae | Depanthus glaber (C.B.Clarke) S.Moore | E | 29 | 1 | 15 | 2.2 | 666 |
| Goodeniaceae | Scaevola balansae Guillaumin | E | 115 | 101 | 19 | 2.8 | 1449 |
| Goodeniaceae | Scaevola cylindrica Schltr. & K.Krause | A | 71 | 53 | 22 | 3.5 | 1309 |
| Goodeniaceae | Scaevola erosa Guillaumin ex I.H.Müller | E | 105 | 101 | 27 | 3.5 | 1198 |
| Goodeniaceae | Scaevola macropyrena I.H.Müller | E | 13 | 13 | 13 | 1.3 | 471 |
| Hernandiaceae | Hernandia cordigera Vieill. | E | 87 | 18 | 64 | 2.9 | 1111 |
| Icacinaceae | Apodytes clusiifolia (Baill.) Villiers | E | 220 | 145 | 96 | 3.5 | 1606 |
| Lamiaceae | Gmelina lignumvitreum Guillaumin | E | 11 | 11 | 1 | 1.8 | 246 |
| Lamiaceae | Gmelina neocaledonica S.Moore | E | 45 | 45 | 3 | 1.3 | 793 |
| Lamiaceae | Vitex sp. "Veillon 7016" | E | 1 | 1 | 0 | 0.0 | 0 |
| Lamiaceae | Vitex collina (Montrouz.) Beauvis. | A | 41 | 34 | 8 | 2.9 | 950 |
| Lamiaceae | Gmelina magnifica Mabb. | E | 22 | 0 | 17 | 2.2 | 921 |
| Lauraceae | Beilschmiedia oreophila Schltr. | E | 46 | 45 | 26 | 2.8 | 1225 |
| Lauraceae | Cryptocarya aristata Kosterm. | E | 39 | 3 | 34 | 3.0 | 1474 |
| Lauraceae | Cryptocarya bitriplinervia Kosterm. | E | 2 | 1 | 2 | 0.3 | 512 |
| Lauraceae | Cryptocarya chartacea Kosterm. | E | 10 | 1 | 6 | 1.5 | 951 |
| Lauraceae | Cryptocarya elliptica Schltr. | E | 67 | 12 | 44 | 3.2 | 851 |
| Lauraceae | Cryptocarya gracilis Schltr. | E | 79 | 57 | 37 | 2.7 | 1451 |
| Lauraceae | Cryptocarya guillauminii Kosterm. | E | 111 | 110 | 32 | 2.6 | 1056 |
| Lauraceae | Cryptocarya longifolia Kosterm. | E | 92 | 42 | 60 | 2.7 | 982 |
| Lauraceae | Cryptocarya mackeei Kosterm. | E | 11 | 11 | 2 | 2.2 | 724 |
| Lauraceae | Cryptocarya macrocarpa Guillaumin | E | 26 | 14 | 18 | 2.6 | 1026 |
| Lauraceae | Cryptocarya macrodesme Schltr. | E | 62 | 4 | 52 | 2.6 | 1174 |
| Lauraceae | Cryptocarya odorata Guillaumin | E | 120 | 102 | 26 | 3.1 | 852 |
| Lauraceae | Cryptocarya oubatchensis Schltr. | E | 97 | 7 | 79 | 3.0 | 1279 |
| Lauraceae | Cryptocarya phyllostemon Kosterm. | E | 31 | 31 | 16 | 2.4 | 854 |
| Lauraceae | Cryptocarya pluricostata Kosterm. | E | 56 | 6 | 33 | 2.0 | 836 |
| Lauraceae | Cryptocarya transversa Kosterm. | E | 105 | 102 | 35 | 2.6 | 1000 |
| Lauraceae | Cryptocarya velutinosa Kosterm. | E | 74 | 3 | 56 | 2.7 | 824 |
| Lauraceae | Endiandra baillonii (Pancher & Sebert) Guillaumin | E | 126 | 116 | 45 | 2.6 | 1538 |
| Lauraceae | Endiandra neocaledonica Kosterm. | E | 15 | 9 | 13 | 2.7 | 784 |
| Lauraceae | Endiandra polyneura Schltr. | E | 13 | 6 | 9 | 2.4 | 641 |
| Lauraceae | Endiandra sebertii Guillaumin | E | 46 | 40 | 3 | 1.9 | 711 |
| Lauraceae | Litsea ripidion Guillaumin | E | 22 | 22 | 4 | 1.6 | 803 |
| Lauraceae | Litsea triflora Guillaumin | E | 92 | 87 | 18 | 3.1 | 1068 |
| Lauraceae | Cryptocarya sp. "Munzinger 4792" | E | 14 | 0 | 14 | 1.5 | 418 |
| Lauraceae | Cryptocarya sp. "Barrabé 280" | E | 23 | 1 | 20 | 2.7 | 821 |
| Lauraceae | Cryptocarya sp. "Munzinger 5874" | E | 29 | 2 | 23 | 2.7 | 821 |
| Lauraceae | Cryptocarya sp. "McPherson 4408" | E | 9 | 9 | 1 | 0.2 | 211 |
| Lauraceae | Cryptocarya sp. "Munzinger 5832" | E | 20 | 0 | 20 | 1.7 | 707 |
| Lauraceae | Cryptocarya sp. ""Munzinger 5178" | E | 4 | 0 | 0 | 0.7 | 122 |
| Lecythidaceae | Barringtonia longifolia Schltr. | E | 18 | 0 | 16 | 2.5 | 835 |
| Lecythidaceae | Barringtonia neocaledonica Vieill. | E | 10 | 1 | 8 | 1.6 | 886 |
| Linaceae | Hugonia penicillanthemum Baill. ex Pancher & Sebert | E | 82 | 79 | 14 | 2.9 | 588 |
| Loganiaceae | Neuburgia novocaledonica (Gilg & Benedict) J.Molina & Struwe | A | 60 | 12 | 38 | 2.7 | 751 |
| Malpighiaceae | Acridocarpus austrocaledonicus Baill. | E | 125 | 108 | 10 | 2.2 | 813 |
| Malvaceae | Acropogon aoupiniensis Morat | E | 21 | 0 | 19 | 0.6 | 701 |
| Malvaceae | Acropogon austrocaledonicus (Hook.f.) Morat | E | 28 | 27 | 18 | 1.5 | 890 |
| Malvaceae | Acropogon domatifer Morat | E | 6 | 1 | 5 | 0.7 | 535 |
| Malvaceae | Acropogon dzumacensis (Guillaumin) Morat | E | 42 | 41 | 15 | 3.1 | 1203 |
| Malvaceae | Acropogon francii (Guillaumin) Morat | E | 40 | 40 | 16 | 2.1 | 1020 |
| Malvaceae | Acropogon sageniifolius Schltr. | E | 9 | 0 | 5 | 0.8 | 759 |
| Malvaceae | Acropogon scheffleraefolius (Guillaumin) Morat | E | 17 | 12 | 2 | 2.1 | 868 |
| Malvaceae | Acropogon schumannianus Schltr. | E | 41 | 6 | 26 | 2.5 | 1279 |
| Malvaceae | Maxwellia lepidota Baill. | E | 88 | 83 | 9 | 2.8 | 887 |
| Malvaceae | Acropogon merytifolius Morat & Chalopin | E | 29 | 0 | 25 | 1.5 | 969 |
| Malvaceae | Acropogon grandiflorus Morat & Chalopin | E | 15 | 0 | 13 | 1.8 | 735 |
| Malvaceae | Acropogon schistophilus Morat & Chalopin | E | 25 | 0 | 22 | 2.3 | 811 |
| Malvaceae | Acropogon macrocarpus Morat & Chalopin | E | 21 | 0 | 15 | 1.0 | 518 |
| Meliaceae | Anthocarapa nitidula (Benth.) T.D.Penn. ex Mabb. | A | 107 | 11 | 81 | 3.2 | 883 |
| Meliaceae | Dysoxylum bijugum (Labill.) Seem. | A | 39 | 10 | 12 | 2.1 | 764 |
| Meliaceae | Dysoxylum canalense (Baill.) C.DC. | E | 152 | 136 | 17 | 2.4 | 963 |
| Meliaceae | Dysoxylum kouiriense Virot | E | 58 | 1 | 50 | 2.5 | 903 |
| Meliaceae | Dysoxylum macranthum C.DC. | E | 36 | 1 | 24 | 1.8 | 710 |
| Meliaceae | Dysoxylum macrostachyum C.DC. | E | 34 | 26 | 12 | 2.0 | 830 |
| Meliaceae | Dysoxylum minutiflorum C.DC. | E | 65 | 64 | 10 | 2.1 | 663 |
| Meliaceae | Dysoxylum pachypodum (Baill.) C.DC. | E | 2 | 1 | 1 | 0.8 | 439 |
| Meliaceae | Dysoxylum roseum C.DC. | E | 204 | 71 | 142 | 3.5 | 1418 |
| Meliaceae | Dysoxylum rufescens Vieill. ex Pancher & Sebert | E | 274 | 167 | 87 | 3.4 | 1436 |
| Monimiaceae | Hedycarya baudouini Baill. | E | 10 | 4 | 4 | 1.2 | 634 |
| Monimiaceae | Hedycarya chrysophylla Perkins | E | 42 | 0 | 33 | 3.0 | 1232 |
| Monimiaceae | Hedycarya cupulata Baill. | E | 171 | 34 | 129 | 2.8 | 1220 |
| Monimiaceae | Hedycarya engleriana S.Moore | E | 71 | 4 | 56 | 2.7 | 807 |
| Monimiaceae | Hedycarya parvifolia Perkins & Schltr. | E | 161 | 127 | 90 | 3.2 | 1430 |
| Monimiaceae | Hedycarya symplocoides S.Moore | E | 6 | 0 | 5 | 1.7 | 497 |
| Monimiaceae | Kibaropsis caledonica (Guillaumin) Jérémie | E | 84 | 0 | 66 | 2.5 | 802 |
| Moraceae | Ficus auriculigera Bureau | E | 47 | 35 | 13 | 1.5 | 999 |
| Moraceae | Ficus austrocaledonica Bureau | E | 84 | 71 | 38 | 2.7 | 1328 |
| Moraceae | Ficus dzumacensis Guillaumin | E | 50 | 48 | 19 | 2.1 | 783 |
| Moraceae | Ficus habrophylla G.Benn. ex Seem. | A | 31 | 7 | 13 | 2.5 | 961 |
| Moraceae | Ficus leiocarpa (Bureau) Warb. | E | 9 | 1 | 5 | 0.8 | 587 |
| Moraceae | Ficus hillii F.M.Bailey | A | 14 | 4 | 7 | 3.5 | 841 |
| Moraceae | Ficus mutabilis Bureau | E | 17 | 3 | 5 | 1.7 | 1010 |
| Moraceae | Ficus nitidifolia Bureau | E | 66 | 66 | 12 | 2.7 | 1038 |
| Moraceae | Ficus obliqua G.Forst. | A | 12 | 0 | 5 | 1.6 | 536 |
| Moraceae | Ficus otophora Corner & Guillaumin | E | 37 | 1 | 29 | 2.4 | 600 |
| Moraceae | Ficus pancheriana Bureau | E | 24 | 1 | 23 | 1.4 | 651 |
| Moraceae | Ficus racemigera Bureau | E | 96 | 27 | 70 | 2.6 | 1119 |
| Moraceae | Ficus versicolor Bureau | E | 27 | 2 | 17 | 1.8 | 676 |
| Moraceae | Ficus vieillardiana Bureau | E | 106 | 76 | 48 | 2.7 | 991 |
| Moraceae | Ficus webbiana (Miq.) Miq. | E | 75 | 35 | 36 | 2.3 | 1156 |
| Moraceae | Sparattosyce balansae A.G.Richt. ex Guillaumin | E | 18 | 4 | 9 | 1.1 | 756 |
| Moraceae | Sparattosyce dioica Bureau | E | 151 | 128 | 46 | 2.6 | 952 |
| Myodocarpaceae | Delarbrea collina Vieill. | E | 10 | 3 | 3 | 1.8 | 506 |
| Myodocarpaceae | Delarbrea harmsii R.Vig. | E | 37 | 2 | 29 | 2.2 | 810 |
| Myodocarpaceae | Delarbrea longicarpa R.Vig. | E | 30 | 28 | 6 | 1.3 | 577 |
| Myodocarpaceae | Delarbrea paradoxa Vieill. | A | 36 | 20 | 7 | 3.1 | 768 |
| Myodocarpaceae | Myodocarpus crassifolius Dubard & R.Vig. | E | 63 | 63 | 19 | 3.8 | 1460 |
| Myodocarpaceae | Myodocarpus fraxinifolius Brongn. & Gris | E | 254 | 234 | 43 | 2.9 | 1532 |
| Myodocarpaceae | Myodocarpus gracilis (Dubard & R.Vig.) Lowry | E | 24 | 23 | 15 | 2.9 | 1383 |
| Myodocarpaceae | Myodocarpus involucratus Dubard & R.Vig. | E | 121 | 117 | 24 | 2.6 | 999 |
| Myodocarpaceae | Myodocarpus nervatus Lowry, ined. | E | 41 | 41 | 5 | 0.9 | 673 |
| Myodocarpaceae | Myodocarpus pinnatus Brongn. & Gris | E | 71 | 8 | 41 | 3.0 | 829 |
| Myodocarpaceae | Myodocarpus simplicifolius Brongn. & Gris | E | 13 | 0 | 9 | 1.5 | 568 |
| Myodocarpaceae | Myodocarpus vieillardii Brongn. & Gris | E | 106 | 85 | 14 | 2.2 | 1005 |
| Myrtaceae | Archirhodomyrtus baladensis (Brongn. & Gris) Burret | E | 48 | 34 | 15 | 2.2 | 1270 |
| Myrtaceae | Archirhodomyrtus paitensis (Schltr.) Burret | E | 15 | 14 | 2 | 1.8 | 936 |
| Myrtaceae | Archirhodomyrtus turbinata (Schltr.) Burret | E | 59 | 58 | 11 | 3.0 | 1257 |
| Myrtaceae | Archirhodomyrtus vieillardii (Brongn. & Gris) Burret | E | 3 | 3 | 2 | 1.7 | 297 |
| Myrtaceae | Arillastrum gummiferum (Pancher ex Brongn. & Gris) Baill. | E | 91 | 82 | 7 | 2.6 | 623 |
| Myrtaceae | Carpolepis laurifolia (Brongn. & Gris) J.W.Dawson | E | 173 | 124 | 103 | 3.4 | 1490 |
| Myrtaceae | Carpolepis tardiflora J.W.Dawson | E | 39 | 23 | 32 | 2.7 | 1175 |
| Myrtaceae | Cloezia floribunda Brongn. & Gris | E | 99 | 74 | 20 | 3.2 | 1277 |
| Myrtaceae | Eugenia brongniartiana Guillaumin | E | 87 | 65 | 21 | 3.4 | 943 |
| Myrtaceae | Eugenia gacognei Montrouz. | E | 32 | 19 | 1 | 2.3 | 687 |
| Myrtaceae | Eugenia paludosa Pancher ex Brongn. & Gris | E | 12 | 4 | 7 | 1.0 | 441 |
| Myrtaceae | Melaleuca quinquenervia (Cav.) S.T.Blake | A | 66 | 21 | 22 | 2.7 | 1195 |
| Myrtaceae | Metrosideros brevistylis J.W.Dawson | E | 75 | 42 | 70 | 3.0 | 1085 |
| Myrtaceae | Metrosideros microphylla (Schltr.) J.W.Dawson | E | 47 | 46 | 46 | 2.9 | 1058 |
| Myrtaceae | Metrosideros nitida Brongn. & Gris | E | 95 | 79 | 37 | 2.8 | 1433 |
| Myrtaceae | Metrosideros oreomyrtus Däniker | E | 13 | 8 | 13 | 2.4 | 477 |
| Myrtaceae | Metrosideros punctata J.W.Dawson | E | 66 | 64 | 27 | 3.2 | 1197 |
| Myrtaceae | Piliocalyx bullatus Brongn. & Gris | E | 34 | 3 | 29 | 2.4 | 618 |
| Myrtaceae | Piliocalyx francii Guillaumin | E | 21 | 15 | 13 | 2.8 | 882 |
| Myrtaceae | Piliocalyx laurifolius Brongn. & Gris | E | 82 | 54 | 30 | 2.5 | 920 |
| Myrtaceae | Piliocalyx wagapensis Brongn. & Gris | E | 65 | 4 | 47 | 2.6 | 1070 |
| Myrtaceae | Pleurocalyptus austrocaledonicus (Guillaumin) J.W.Dawson | E | 7 | 4 | 2 | 1.2 | 599 |
| Myrtaceae | Pleurocalyptus pancheri (Brongn. & Gris) J.W.Dawson | E | 130 | 127 | 45 | 2.7 | 981 |
| Myrtaceae | Rhodamnia andromedoides Guillaumin | E | 53 | 53 | 9 | 2.1 | 872 |
| Myrtaceae | Rhodomyrtus locellata (Guillaumin) Burret | E | 51 | 50 | 14 | 2.4 | 894 |
| Myrtaceae | Syzygium acre (Pancher ex Guillaumin) J.W.Dawson | E | 7 | 6 | 2 | 1.2 | 295 |
| Myrtaceae | Syzygium amieuense (Guillaumin) J.W.Dawson | E | 30 | 1 | 24 | 2.5 | 522 |
| Myrtaceae | Syzygium aoupinianum J.W.Dawson | E | 20 | 2 | 17 | 0.8 | 657 |
| Myrtaceae | Syzygium apetiolatum J.W.Dawson | E | 6 | 0 | 5 | 0.5 | 267 |
| Myrtaceae | Syzygium arboreum (Baker f.) J.W.Dawson | E | 49 | 16 | 32 | 3.3 | 1481 |
| Myrtaceae | Syzygium auriculatum Brongn. & Gris | E | 25 | 0 | 18 | 3.0 | 498 |
| Myrtaceae | Syzygium austrocaledonicum (Seem.) Guillaumin | E | 79 | 74 | 12 | 2.9 | 998 |
| Myrtaceae | Syzygium baladense (Brongn. & Gris) J.W.Dawson | E | 56 | 47 | 17 | 2.9 | 927 |
| Myrtaceae | Syzygium balansae (Guillaumin) J.W.Dawson | E | 16 | 4 | 6 | 3.2 | 1289 |
| Myrtaceae | Syzygium brachycalyx (Baker f.) J.W.Dawson | E | 9 | 1 | 8 | 2.0 | 595 |
| Myrtaceae | Syzygium brongniartii (Merr. & L.M.Perry) J.W.Dawson | E | 87 | 72 | 46 | 2.8 | 1025 |
| Myrtaceae | Syzygium capillaceum (Brongn. & Gris) J.W.Dawson | E | 21 | 10 | 7 | 2.1 | 898 |
| Myrtaceae | Syzygium coccineum J.W.Dawson | E | 29 | 12 | 22 | 2.7 | 1126 |
| Myrtaceae | Syzygium conceptionis Guillaumin | E | 9 | 9 | 5 | 1.1 | 814 |
| Myrtaceae | Syzygium densiflorum Brongn. & Gris | E | 37 | 23 | 13 | 2.6 | 850 |
| Myrtaceae | Syzygium deplanchei (Guillaumin) J.W.Dawson | E | 54 | 32 | 36 | 3.0 | 1418 |
| Myrtaceae | Syzygium frutescens Brongn. & Gris | E | 147 | 129 | 36 | 3.1 | 1102 |
| Myrtaceae | Syzygium guillauminii J.W.Dawson | E | 22 | 1 | 16 | 1.9 | 1121 |
| Myrtaceae | Syzygium longifolium (Brongn. & Gris) J.W.Dawson | E | 9 | 3 | 5 | 2.0 | 885 |
| Myrtaceae | Syzygium macranthum Brongn. & Gris | E | 153 | 117 | 46 | 3.6 | 1345 |
| Myrtaceae | Syzygium meorianum J.W.Dawson | E | 2 | 2 | 0 | 0.5 | 478 |
| Myrtaceae | Syzygium mouanum Guillaumin | E | 68 | 60 | 28 | 3.3 | 1549 |
| Myrtaceae | Syzygium multipetalum Pancher ex Brongn. & Gris | E | 121 | 114 | 24 | 2.9 | 1374 |
| Myrtaceae | Syzygium neocaledonicum (Seem.) J.W.Dawson | E | 16 | 0 | 10 | 3.0 | 594 |
| Myrtaceae | Syzygium pancheri Brongn. & Gris | E | 107 | 85 | 34 | 3.5 | 1334 |
| Myrtaceae | Syzygium paniense (Baker f.) J.W.Dawson | E | 33 | 0 | 32 | 2.5 | 804 |
| Myrtaceae | Syzygium quadrangulare Guillaumin | E | 27 | 22 | 20 | 2.6 | 972 |
| Myrtaceae | Syzygium rhopalanthum Schltr. | E | 59 | 58 | 35 | 3.3 | 1293 |
| Myrtaceae | Syzygium schlechterianum Hochr. | E | 6 | 0 | 3 | 2.1 | 918 |
| Myrtaceae | Syzygium tenuiflorum Brongn. & Gris | E | 16 | 5 | 9 | 2.0 | 726 |
| Myrtaceae | Syzygium toninense (Baker f.) J.W.Dawson | E | 29 | 2 | 25 | 1.5 | 717 |
| Myrtaceae | Syzygium tripetalum Guillaumin | E | 49 | 23 | 40 | 2.5 | 1420 |
| Myrtaceae | Syzygium wagapense Brongn. & Gris | E | 49 | 35 | 8 | 2.0 | 903 |
| Myrtaceae | Syzygium xanthostemifolium (Guillaumin) J.W.Dawson | E | 11 | 11 | 4 | 1.7 | 880 |
| Myrtaceae | Tristaniopsis capitulata Brongn. & Gris | E | 14 | 13 | 2 | 2.5 | 527 |
| Myrtaceae | Tristaniopsis guillainii Vieill. ex Brongn. & Gris | E | 228 | 211 | 35 | 3.4 | 1152 |
| Myrtaceae | Tristaniopsis reticulata J.W.Dawson | E | 30 | 28 | 4 | 2.0 | 554 |
| Myrtaceae | Uromyrtus ngoyensis (Schltr.) Burret | E | 84 | 76 | 21 | 3.3 | 1542 |
| Myrtaceae | Xanthostemon aurantiacus (Brongn. & Gris) Schltr. | E | 14 | 14 | 1 | 1.5 | 690 |
| Myrtaceae | Xanthostemon ruber (Brongn. & Gris) Sebert & Pancher | E | 31 | 31 | 5 | 2.4 | 720 |
| Myrtaceae | Xanthostemon velutinus (Gugerli) J.W.Dawson | E | 10 | 7 | 2 | 1.1 | 407 |
| Myrtaceae | Piliocalyx ignambiensis (Baker f.) Craven, comb. ined. | E | 3 | 0 | 3 | 0.9 | 46 |
| Myrtaceae | Gossia vieillardii (Brongn. & Gris) N.Snow | A | 84 | 63 | 25 | 2.6 | 1210 |
| Myrtaceae | Kanakomyrtus myrtopsidoides Guillaumin ex N.Snow | E | 13 | 13 | 11 | 1.7 | 769 |
| Myrtaceae | Kanakomyrtus longipetiolata N.Snow | E | 14 | 0 | 8 | 2.5 | 780 |
| Myrtaceae | Kanakomyrtus prominens N.Snow | E | 11 | 0 | 11 | 1.6 | 490 |
| Myrtaceae | Gossia pancheri (Brongn. & Gris) N.Snow | E | 119 | 115 | 22 | 3.2 | 1330 |
| Myrtaceae | Gossia nigripes (Guillaumin) N.Snow | E | 3 | 1 | 1 | 2.5 | 515 |
| Myrtaceae | Gossia clusioides (Brongn. & Gris) N.Snow | E | 79 | 61 | 24 | 3.2 | 1409 |
| Myrtaceae | Eugenia ovigera Brongn. & Gris | E | 15 | 10 | 1 | 2.1 | 565 |
| Myrtaceae | Eugenia rubiginosa (Brongn. & Gris) J.W.Dawson, comb. IMPOSSIBLE | E | 25 | 24 | 5 | 3.3 | 995 |
| Myrtaceae | Eugenia poroensis J.W.Dawson, ined. | E | 39 | 36 | 0 | 1.6 | 436 |
| Myrtaceae | Piliocalyx sp. "MacKee 26643" | E | 3 | 0 | 3 | 0.3 | 301 |
| Myrtaceae | Xanthomyrtus kanalaensis (Hochr.) N.Snow | E | 58 | 55 | 22 | 2.7 | 941 |
| Nothofagaceae | Nothofagus aequilateralis (Baum.-Bod.) Steenis | E | 51 | 45 | 12 | 1.7 | 1117 |
| Nothofagaceae | Nothofagus balansae (Baill.) Steenis | E | 38 | 38 | 12 | 2.4 | 1248 |
| Nyctaginaceae | Pisonia artensis (Montrouz.) Heimerl | E | 4 | 2 | 1 | 1.1 | 505 |
| Nyctaginaceae | Pisonia gigantocarpa (Heimerl) Stemm. | E | 63 | 13 | 46 | 2.9 | 897 |
| Olacaceae | Olax hypoleuca Baill. | E | 58 | 52 | 9 | 2.6 | 715 |
| Oleaceae | Chionanthus brachystachys (Schltr.) P.S.Green | E | 186 | 99 | 89 | 3.2 | 1218 |
| Oleaceae | Chionanthus pedunculatus P.S.Green | E | 44 | 1 | 34 | 2.2 | 955 |
| Oleaceae | Olea paniculata R.Br. | A | 12 | 2 | 4 | 1.7 | 583 |
| Oleaceae | Osmanthus austrocaledonicus (Vieill.) Knobl. | E | 273 | 228 | 42 | 3.9 | 1598 |
| Oncothecaceae | Oncotheca balansae Baill. | E | 53 | 49 | 8 | 2.8 | 551 |
| Oncothecaceae | Oncotheca humboldtiana (Guillaumin) Morat & Veillon | E | 56 | 52 | 18 | 3.0 | 973 |
| Pandanaceae | Pandanus altissimus (Brongn.) Solms | E | 26 | 21 | 18 | 3.4 | 1295 |
| Pandanaceae | Pandanus balansae (Brongn.) Solms | E | 60 | 53 | 21 | 2.5 | 881 |
| Pandanaceae | Pandanus bernardii H.St.John ex Callm. ined. | E | 36 | 36 | 4 | 1.4 | 834 |
| Pandanaceae | Pandanus clandestinus Stone | E | 11 | 0 | 11 | 1.3 | 432 |
| Pandanaceae | Pandanus pancheri (Brongn.) Solms | E | 40 | 39 | 8 | 1.4 | 1013 |
| Pandanaceae | Pandanus bilinearis H.St.John | E | 12 | 8 | 3 | 1.4 | 547 |
| Paracryphiaceae | Paracryphia alticola (Schltr.) Steenis | E | 44 | 23 | 39 | 2.7 | 1248 |
| Paracryphiaceae | Quintinia major (Baill.) Schltr. | E | 50 | 50 | 37 | 2.6 | 1123 |
| Paracryphiaceae | Quintinia oreophila (Schltr.) Schltr. | E | 21 | 21 | 21 | 2.2 | 992 |
| Paracryphiaceae | Quintinia parviflora (Schltr.) Schltr. | E | 9 | 2 | 6 | 2.5 | 1003 |
| Paracryphiaceae | Quintinia resinosa (Schltr.) Schltr. | E | 13 | 12 | 8 | 2.0 | 1077 |
| Paracryphiaceae | Sphenostemon comptonii Baker f. | E | 10 | 0 | 10 | 1.6 | 1011 |
| Paracryphiaceae | Sphenostemon pachycladum Baill. | E | 70 | 65 | 33 | 3.4 | 1356 |
| Paracryphiaceae | Sphenostemon thibaudii Jérémie | E | 2 | 0 | 2 | 0.9 | 200 |
| Phellinaceae | Phelline billardierei Pancher ex Loes. | E | 4 | 4 | 0 | 0.4 | 213 |
| Phellinaceae | Phelline brachyphylla Baill. | E | 26 | 5 | 16 | 2.5 | 923 |
| Phellinaceae | Phelline comosa Labill. | E | 77 | 60 | 46 | 3.5 | 1289 |
| Phellinaceae | Phelline confertifolia Baill. | E | 79 | 51 | 58 | 2.9 | 1358 |
| Phellinaceae | Phelline dumbeensis Guillaumin | E | 64 | 18 | 39 | 2.0 | 1251 |
| Phellinaceae | Phelline erubescens Baill. | E | 29 | 2 | 17 | 2.6 | 1152 |
| Phellinaceae | Phelline lucida Vieill. ex Baill. | E | 119 | 117 | 57 | 3.5 | 1459 |
| Phellinaceae | Phelline macrophylla Baill. | E | 23 | 22 | 8 | 2.2 | 938 |
| Phyllanthaceae | Bischofia javanica Blume | A | 16 | 1 | 6 | 1.0 | 748 |
| Phyllanthaceae | Cleistanthus stipitatus (Baill.) Müll.Arg. | E | 106 | 76 | 16 | 2.7 | 849 |
| Phyllanthaceae | Phyllanthus billardierei (Baill.) Müll.Arg. | E | 87 | 11 | 51 | 3.2 | 1178 |
| Phyllanthaceae | Phyllanthus caledonicus (Müll.Arg.) Müll.Arg. | E | 10 | 2 | 3 | 1.7 | 719 |
| Picrodendraceae | Austrobuxus alticola McPherson | E | 19 | 4 | 18 | 2.3 | 1302 |
| Picrodendraceae | Austrobuxus brevipes AiryShaw | E | 62 | 60 | 27 | 3.7 | 1366 |
| Picrodendraceae | Austrobuxus carunculatus (Baill.) Airy Shaw | E | 150 | 114 | 22 | 3.4 | 1283 |
| Picrodendraceae | Austrobuxus cuneatus (AiryShaw) Airy Shaw | A | 80 | 80 | 6 | 2.6 | 955 |
| Picrodendraceae | Austrobuxus eugeniifolius (Guillaumin) Airy Shaw | E | 43 | 42 | 14 | 2.4 | 1045 |
| Picrodendraceae | Austrobuxus huerlimannii AiryShaw | E | 28 | 28 | 2 | 1.2 | 554 |
| Picrodendraceae | Austrobuxus ovalis AiryShaw | E | 19 | 1 | 17 | 1.9 | 815 |
| Picrodendraceae | Austrobuxus pauciflorus AiryShaw | E | 67 | 65 | 13 | 2.2 | 1233 |
| Picrodendraceae | Austrobuxus rubiginosus (Guillaumin) Airy Shaw | E | 42 | 42 | 3 | 1.9 | 400 |
| Picrodendraceae | Austrobuxus vieillardii (Guillaumin) Airy Shaw | E | 34 | 2 | 27 | 2.3 | 1211 |
| Picrodendraceae | Scagea depauperata (Baill.) McPherson | E | 34 | 32 | 2 | 2.0 | 566 |
| Pittosporaceae | Pittosporum aliferum Tirel & Veillon | E | 8 | 7 | 2 | 3.3 | 1154 |
| Pittosporaceae | Pittosporum deplanchei Brongn. & Gris | E | 96 | 92 | 25 | 2.4 | 866 |
| Pittosporaceae | Pittosporum gracile Pancher ex Brongn. & Gris | E | 85 | 63 | 17 | 2.5 | 995 |
| Pittosporaceae | Pittosporum heckelii Dubard | E | 34 | 13 | 18 | 3.0 | 1412 |
| Pittosporaceae | Pittosporum hematomallum Guillaumin | E | 30 | 29 | 4 | 1.6 | 532 |
| Pittosporaceae | Pittosporum leratii Guillaumin | E | 34 | 34 | 17 | 2.8 | 981 |
| Pittosporaceae | Pittosporum letocartiorum Veillon & Tirel | E | 12 | 10 | 5 | 1.2 | 983 |
| Pittosporaceae | Pittosporum oreophilum Guillaumin | E | 38 | 1 | 28 | 2.6 | 1056 |
| Pittosporaceae | Pittosporum oubatchense Schltr. | E | 21 | 21 | 21 | 2.0 | 1270 |
| Pittosporaceae | Pittosporum paniense Guillaumin | E | 8 | 0 | 8 | 1.0 | 704 |
| Pittosporaceae | Pittosporum pronyense Guillaumin | E | 109 | 107 | 42 | 3.2 | 1216 |
| Pittosporaceae | Pittosporum simsonii Montrouz. | E | 31 | 6 | 8 | 1.6 | 932 |
| Pittosporaceae | Pittosporum xanthanthum Schltr. | E | 53 | 52 | 42 | 3.1 | 1159 |
| Podocarpaceae | Dacrydium araucarioides Brongn. & Gris | E | 94 | 91 | 9 | 2.8 | 982 |
| Podocarpaceae | Falcatifolium taxoides (Brongn. & Gris) de Laub. | E | 74 | 62 | 45 | 3.2 | 1141 |
| Podocarpaceae | Podocarpus lucienii de Laub. | E | 97 | 85 | 22 | 2.6 | 1511 |
| Podocarpaceae | Podocarpus sylvestris J.Buchholz | E | 60 | 30 | 28 | 2.8 | 1120 |
| Podocarpaceae | Prumnopitys ferruginoides (Compton) de Laub. | E | 21 | 16 | 18 | 2.3 | 1392 |
| Podocarpaceae | Retrophyllum comptonii (J.Buchholz) C.N.Page | E | 108 | 89 | 76 | 3.2 | 1569 |
| Primulaceae | Tapeinosperma deflexum Mez | E | 24 | 22 | 20 | 3.0 | 834 |
| Primulaceae | Tapeinosperma glandulosum Guillaumin | E | 33 | 3 | 31 | 2.3 | 1009 |
| Primulaceae | Tapeinosperma gracile Mez | E | 77 | 25 | 55 | 3.0 | 1062 |
| Primulaceae | Tapeinosperma minutum Mez | E | 16 | 0 | 10 | 2.5 | 1510 |
| Primulaceae | Tapeinosperma nectandroides Mez | E | 14 | 8 | 8 | 1.1 | 607 |
| Primulaceae | Tapeinosperma nitidum Mez | E | 23 | 0 | 20 | 2.7 | 1478 |
| Primulaceae | Tapeinosperma oblongifolium Mez | E | 22 | 2 | 16 | 1.6 | 853 |
| Primulaceae | Tapeinosperma pancheri Mez | E | 56 | 17 | 50 | 2.4 | 873 |
| Primulaceae | Tapeinosperma robustum Mez | E | 75 | 66 | 30 | 3.3 | 1339 |
| Primulaceae | Tapeinosperma rubidum Mez | E | 16 | 2 | 15 | 2.2 | 396 |
| Primulaceae | Tapeinosperma vestitum Mez | E | 26 | 25 | 22 | 2.9 | 1245 |
| Primulaceae | Tapeinosperma vieillardii Hook.f. | E | 69 | 16 | 50 | 2.7 | 1035 |
| Primulaceae | Tapeinosperma wagapense Mez | E | 26 | 2 | 20 | 1.6 | 638 |
| Primulaceae | Tapeinosperma scrobiculatum (Seem.) Mez | A | 14 | 0 | 11 | 2.6 | 397 |
| Primulaceae | Myrsine arborea (M.Schmid) Ricketson & Pipoly | E | 31 | 1 | 19 | 2.4 | 662 |
| Primulaceae | Myrsine asymmetrica (Mez) Ricketson & Pipoly | E | 236 | 201 | 85 | 3.3 | 1546 |
| Primulaceae | Myrsine citrifolia (Mez) Ricketson & Pipoly | E | 36 | 6 | 23 | 3.3 | 1100 |
| Primulaceae | Myrsine diminuta (Mez) Ricketson & Pipoly | E | 67 | 64 | 23 | 3.4 | 1443 |
| Primulaceae | Myrsine discocarpa (M.Schmid) Ricketson & Pipoly | E | 20 | 6 | 5 | 1.9 | 778 |
| Primulaceae | Myrsine lanceolata Pancher & Sebert | E | 55 | 48 | 12 | 2.1 | 1040 |
| Primulaceae | Myrsine macrophylla (Mez) Ricketson & Pipoly | E | 49 | 48 | 16 | 3.6 | 1598 |
| Primulaceae | Myrsine memaoyaensis (M.Schmid) Ricketson & Pipoly | E | 5 | 3 | 5 | 0.6 | 715 |
| Primulaceae | Myrsine modesta (Mez) Ricketson & Pipoly | A | 84 | 53 | 46 | 3.1 | 1599 |
| Primulaceae | Myrsine novocaledonica (Mez) Ricketson & Pipoly | E | 45 | 30 | 7 | 3.2 | 1062 |
| Primulaceae | Myrsine oblanceolata (M.Schmid) Ricketson & Pipoly | E | 46 | 42 | 4 | 1.5 | 811 |
| Primulaceae | Myrsine ovicarpa (M.Schmid) Ricketson & Pipoly | E | 22 | 22 | 0 | 0.5 | 306 |
| Primulaceae | Myrsine parvicarpa (M.Schmid) Ricketson & Pipoly | E | 13 | 11 | 13 | 2.2 | 1030 |
| Primulaceae | Tapeinosperma poueboense M.Schmid | E | 11 | 0 | 7 | 2.3 | 1005 |
| Proteaceae | Beauprea asplenioides Schltr. | E | 10 | 10 | 6 | 1.5 | 992 |
| Proteaceae | Beauprea comptonii S.Moore | E | 16 | 1 | 13 | 2.2 | 1414 |
| Proteaceae | Beauprea filipes Schltr. | E | 25 | 10 | 17 | 2.6 | 739 |
| Proteaceae | Beauprea montana (Brongn. & Gris) Virot | E | 79 | 76 | 15 | 2.9 | 999 |
| Proteaceae | Beauprea pancheri Brongn. & Gris | E | 41 | 39 | 35 | 3.0 | 1273 |
| Proteaceae | Beauprea spathulaefolia Brongn. & Gris | E | 43 | 38 | 16 | 3.5 | 1224 |
| Proteaceae | Garnieria spathulaefolia (Brongn. & Gris) Brongn. & Gris | E | 36 | 34 | 7 | 2.2 | 819 |
| Proteaceae | Grevillea gillivrayi Hook. & Arn. | E | 127 | 103 | 14 | 2.6 | 1381 |
| Proteaceae | Kermadecia rotundifolia Brongn. & Gris | E | 51 | 3 | 40 | 2.7 | 1322 |
| Proteaceae | Kermadecia sinuata Brongn. & Gris | E | 58 | 1 | 42 | 2.3 | 841 |
| Proteaceae | Sleumerodendron austrocaledonicum (Brongn. & Gris) Virot | E | 48 | 7 | 41 | 2.5 | 1295 |
| Proteaceae | Stenocarpus phyllodineus S.Moore | E | 25 | 21 | 5 | 2.6 | 960 |
| Proteaceae | Stenocarpus trinervis (Montrouz.) Guillaumin | E | 177 | 150 | 30 | 3.2 | 1056 |
| Proteaceae | Virotia francii (Guillaumin) P.H.Weston & A.R.Mast | E | 28 | 23 | 9 | 2.0 | 741 |
| Proteaceae | Virotia leptophylla (Guillaumin) L.A.S.Johnson & B.G.Briggs | E | 30 | 9 | 22 | 1.4 | 848 |
| Proteaceae | Virotia neurophylla (Guillaumin) P.H.Weston & A.R.Mast | E | 25 | 24 | 7 | 1.5 | 524 |
| Proteaceae | Virotia rousselii (Vieill.) P.H.Weston & A.R.Mast | E | 16 | 1 | 12 | 1.3 | 803 |
| Rhamnaceae | Alphitonia neocaledonica (Schltr.) Guillaumin | E | 247 | 222 | 26 | 2.5 | 938 |
| Rhamnaceae | Alphitonia xerocarpa Baill. | E | 67 | 65 | 17 | 3.3 | 1120 |
| Rhizophoraceae | Crossostylis grandiflora Pancher ex Brongn. & Gris | E | 79 | 45 | 37 | 3.2 | 1035 |
| Rhizophoraceae | Crossostylis multiflora Brongn. & Gris ex Pancher & Sebert | E | 93 | 3 | 67 | 2.9 | 968 |
| Rhizophoraceae | Crossostylis seberti Pancher ex Brongn. & Gris | E | 28 | 27 | 1 | 1.5 | 472 |
| Rubiaceae | Aidia congestum (Schltr. & K.Krause) Ridsdale | E | 29 | 3 | 26 | 1.2 | 660 |
| Rubiaceae | Aidia vieillardii (Baill.) Ridsdale | E | 20 | 2 | 12 | 1.7 | 731 |
| Rubiaceae | Antirhea rhamnoides (Baill.) Chaw | E | 33 | 30 | 6 | 2.7 | 559 |
| Rubiaceae | Atractocarpus heterophyllus (Montrouz.) Guillaumin & Beauvis. | E | 40 | 34 | 6 | 3.3 | 1105 |
| Rubiaceae | Gardenia aubryi Vieill. | E | 166 | 161 | 36 | 2.5 | 638 |
| Rubiaceae | Guettarda baladensis Guillaumin | E | 60 | 1 | 55 | 2.7 | 1166 |
| Rubiaceae | Guettarda eximia Baill. | E | 153 | 151 | 29 | 2.1 | 992 |
| Rubiaceae | Guettarda glabrescens (Schltr.) Guillaumin | E | 49 | 42 | 8 | 2.6 | 1061 |
| Rubiaceae | Guettarda heterosepala Guillaumin | E | 65 | 58 | 38 | 2.6 | 1088 |
| Rubiaceae | Guettarda ngoyensis (Schltr.) Guillaumin | E | 39 | 37 | 17 | 1.8 | 924 |
| Rubiaceae | Guettarda splendens Baill. | E | 44 | 41 | 5 | 2.1 | 620 |
| Rubiaceae | Guettarda wagapensis Guillaumin | E | 61 | 31 | 33 | 2.4 | 766 |
| Rubiaceae | Ixora cauliflora Montrouz. | E | 57 | 43 | 7 | 2.8 | 860 |
| Rubiaceae | Ixora collina (Montrouz.) Beauvis. | A | 21 | 16 | 3 | 2.4 | 840 |
| Rubiaceae | Ixora comptonii S.Moore | E | 55 | 26 | 38 | 2.6 | 839 |
| Rubiaceae | Ixora francii Schltr. & K.Krause | E | 241 | 217 | 39 | 2.7 | 997 |
| Rubiaceae | Morierina montana Vieill. | E | 16 | 15 | 7 | 1.5 | 418 |
| Rubiaceae | Psychotria collina Labill. | E | 137 | 23 | 90 | 3.1 | 1284 |
| Rubiaceae | Gardenia oudiepe Vieill. | E | 75 | 10 | 53 | 2.8 | 965 |
| Rubiaceae | Gardenia colnettiana Guillaumin | E | 3 | 0 | 3 | 0.1 | 326 |
| Rubiaceae | Gardenia mollis Schltr. | E | 6 | 0 | 1 | 0.9 | 543 |
| Rubiaceae | Randia ngoyensis (Schltr.) Hutch. ex Moore | E | 22 | 20 | 9 | 1.8 | 602 |
| Rubiaceae | Randia pseudoterminalis Guillaumin | E | 32 | 29 | 15 | 2.2 | 876 |
| Rubiaceae | Randia nigricans Schltr. | E | 8 | 0 | 8 | 0.5 | 69 |
| Rubiaceae | Gea crassifolia Achille, ined. | E | 3 | 3 | 1 | 1.0 | 611 |
| Rubiaceae | Atractocarpus sp. "MacKee 25341" | E | 2 | 0 | 2 | 0.7 | 11 |
| Rubiaceae | Guettarda sp. "Munzinger 1414" | E | 1 | 0 | 1 | 0.0 | 0 |
| Rubiaceae | Gynochthodes billardierei (Baill.) Razafim. & B.Bremer | E | 26 | 0 | 20 | 1.7 | 784 |
| Rutaceae | Acronychia laevis J.R.Forst. & G.Forst. | A | 44 | 24 | 10 | 2.6 | 759 |
| Rutaceae | Comptonella drupacea (Labill.) Guillaumin | E | 160 | 106 | 78 | 3.9 | 1623 |
| Rutaceae | Comptonella lactea (Baker f.) T.G.Hartley | E | 84 | 82 | 27 | 2.9 | 1223 |
| Rutaceae | Comptonella microcarpa (Perkins) T.G.Hartley | E | 55 | 27 | 22 | 2.9 | 1287 |
| Rutaceae | Comptonella oreophila (Guillaumin) T.G.Hartley | E | 105 | 56 | 79 | 3.3 | 1436 |
| Rutaceae | Comptonella sessilifoliola (Guillaumin) T.G.Hartley | E | 85 | 64 | 48 | 2.7 | 1204 |
| Rutaceae | Dutaillyea amosensis (Guillaumin) T.G.Hartley | E | 7 | 0 | 5 | 2.3 | 540 |
| Rutaceae | Flindersia fournieri Pancher & Sebert | E | 142 | 137 | 16 | 3.3 | 837 |
| Rutaceae | Geijera balansae (Baill.) Schinz & Guillaumin | E | 6 | 4 | 2 | 0.4 | 488 |
| Rutaceae | Halfordia kendac (Montrouz.) Guillaumin | A | 87 | 79 | 4 | 2.6 | 798 |
| Rutaceae | Melicope lasioneura (Baill.) Baill. ex Guillaumin | E | 71 | 59 | 21 | 2.4 | 1272 |
| Rutaceae | Melicope vieillardii (Baill.) Baill. ex Guillaumin | E | 58 | 43 | 28 | 2.9 | 1436 |
| Rutaceae | Myrtopsis myrtoidea (Baill.) Guillaumin | E | 30 | 22 | 7 | 3.0 | 1135 |
| Rutaceae | Sarcomelicope argyrophylla Guillaumin | E | 58 | 58 | 16 | 2.6 | 755 |
| Rutaceae | Sarcomelicope follicularis T.G.Hartley | E | 24 | 4 | 16 | 2.3 | 806 |
| Rutaceae | Sarcomelicope leiocarpa (P.S.Green) T.G.Hartley | E | 10 | 6 | 1 | 2.0 | 504 |
| Rutaceae | Zanthoxylum albiflorum Baker f. | E | 12 | 11 | 3 | 1.4 | 852 |
| Rutaceae | Zanthoxylum neocaledonicum Baker f. | E | 11 | 1 | 9 | 1.3 | 690 |
| Rutaceae | Zanthoxylum sarasinii Guillaumin | E | 16 | 15 | 3 | 2.1 | 906 |
| Rutaceae | Zanthoxylum schlechteri Guillaumin | E | 8 | 5 | 6 | 1.6 | 703 |
| Rutaceae | Picrella glandulosa T.G.Hartley | E | 29 | 4 | 22 | 1.9 | 1066 |
| Rutaceae | Picrella ignambiensis (Guillaumin) T.G.Hartley & Mabb. | E | 33 | 1 | 17 | 2.2 | 861 |
| Salicaceae | Casearia puberula Guillaumin | E | 60 | 51 | 21 | 2.9 | 802 |
| Salicaceae | Casearia silvana Schltr. | E | 168 | 131 | 33 | 3.0 | 895 |
| Salicaceae | Homalium deplanchei (Vieill.) Warb. | E | 151 | 135 | 21 | 3.5 | 1026 |
| Salicaceae | Homalium guillainii (Vieill.) Briq. | E | 43 | 43 | 2 | 1.5 | 411 |
| Salicaceae | Lasiochlamys cordifolia Sleumer | E | 8 | 1 | 6 | 1.3 | 669 |
| Salicaceae | Lasiochlamys koghiensis (Guillaumin) Sleumer | E | 42 | 42 | 18 | 2.5 | 1020 |
| Salicaceae | Lasiochlamys planchonellifolia (Guillaumin) Sleumer | E | 81 | 80 | 12 | 2.5 | 1206 |
| Salicaceae | Lasiochlamys reticulata (Schltr.) Pax & K.Hoffm. | E | 18 | 0 | 14 | 2.5 | 807 |
| Salicaceae | Lasiochlamys rivularis Sleumer | E | 8 | 8 | 2 | 0.9 | 365 |
| Salicaceae | Xylosma vincentii Guillaumin | E | 36 | 29 | 14 | 2.3 | 834 |
| Santalaceae | Amphorogyne celastroides Stauffer & Hürl. | E | 26 | 26 | 11 | 3.3 | 1126 |
| Santalaceae | Amphorogyne spicata Stauffer & Hürl. | E | 36 | 30 | 16 | 3.1 | 1184 |
| Sapindaceae | Arytera chartacea Radlk. | E | 13 | 3 | 3 | 3.4 | 852 |
| Sapindaceae | Arytera lepidota Radlk. | E | 21 | 17 | 2 | 1.7 | 926 |
| Sapindaceae | Arytera neoebudensis (Guillaumin) H.Turner | A | 9 | 0 | 5 | 2.7 | 615 |
| Sapindaceae | Cupaniopsis apiocarpa Radlk. | E | 15 | 4 | 4 | 2.9 | 963 |
| Sapindaceae | Cupaniopsis azantha Radlk. | E | 10 | 0 | 7 | 1.7 | 731 |
| Sapindaceae | Cupaniopsis chytradenia Radlk. | E | 8 | 0 | 7 | 2.6 | 512 |
| Sapindaceae | Cupaniopsis fruticosa Radlk. | E | 62 | 52 | 18 | 2.8 | 984 |
| Sapindaceae | Cupaniopsis grisea Adema | E | 2 | 1 | 1 | 0.1 | 564 |
| Sapindaceae | Cupaniopsis inoplaea Radlk. | E | 49 | 43 | 9 | 2.7 | 1242 |
| Sapindaceae | Cupaniopsis mackeeana Adema | E | 9 | 1 | 8 | 1.6 | 626 |
| Sapindaceae | Cupaniopsis macrocarpa Radlk. | E | 129 | 19 | 91 | 2.8 | 1265 |
| Sapindaceae | Cupaniopsis myrmoctona Radlk. | E | 50 | 5 | 32 | 2.5 | 1226 |
| Sapindaceae | Cupaniopsis oedipoda Radlk. | E | 88 | 54 | 44 | 3.2 | 980 |
| Sapindaceae | Cupaniopsis petiolulata Radlk. | E | 107 | 6 | 60 | 2.7 | 722 |
| Sapindaceae | Cupaniopsis phalacrocarpa Adema | E | 33 | 1 | 26 | 2.6 | 746 |
| Sapindaceae | Cupaniopsis sylvatica Guillaumin | E | 88 | 24 | 56 | 3.1 | 930 |
| Sapindaceae | Elattostachys apetala (Labill.) Radlk. | A | 60 | 14 | 23 | 2.5 | 996 |
| Sapindaceae | Gongrodiscus bilocularis H.Turner | E | 133 | 87 | 69 | 2.5 | 1150 |
| Sapindaceae | Gongrodiscus sufferrugineus Radlk. | E | 21 | 4 | 13 | 1.1 | 586 |
| Sapindaceae | Guioa glauca (Labill.) Radlk. | E | 378 | 283 | 97 | 3.4 | 1299 |
| Sapindaceae | Guioa microsepala Radlk. | E | 75 | 20 | 67 | 2.7 | 1216 |
| Sapindaceae | Guioa ovalis Radlk. | A | 111 | 38 | 66 | 3.3 | 982 |
| Sapindaceae | Guioa villosa Radlk. | E | 200 | 148 | 50 | 3.3 | 1062 |
| Sapindaceae | Harpullia austrocaledonica Baill. | E | 62 | 3 | 44 | 1.9 | 887 |
| Sapindaceae | Podonephelium concolor Radlk. | E | 16 | 2 | 13 | 0.9 | 833 |
| Sapindaceae | Storthocalyx chryseus Radlk. | E | 113 | 65 | 40 | 3.5 | 994 |
| Sapindaceae | Storthocalyx leioneurus Radlk. | E | 38 | 31 | 10 | 3.2 | 707 |
| Sapindaceae | Storthocalyx pancheri (Baill.) Radlk. | E | 61 | 60 | 10 | 2.3 | 801 |
| Sapindaceae | Storthocalyx sordidus Radlk. | E | 13 | 0 | 9 | 1.4 | 728 |
| Sapindaceae | Podonephelium pachycaule Munzinger, Lowry, Callm. & Buerki | E | 19 | 2 | 10 | 1.8 | 538 |
| Sapindaceae | Podonephelium gongrocarpum (Radlk.) Munzinger, Lowry, Callm. & Buerki | E | 12 | 12 | 1 | 0.2 | 264 |
| Sapotaceae | Planchonella kuebiniensis Aubrév. | E | 116 | 112 | 19 | 3.0 | 935 |
| Sapotaceae | Planchonella thiensis Aubrév. | E | 71 | 67 | 11 | 1.6 | 552 |
| Sapotaceae | Pycnandra benthamii Baill. | E | 26 | 0 | 21 | 2.2 | 874 |
| Sapotaceae | Pycnandra carinocostata Vink | E | 23 | 22 | 16 | 2.6 | 1237 |
| Sapotaceae | Pycnandra chartacea Vink | E | 12 | 11 | 2 | 0.9 | 438 |
| Sapotaceae | Pycnandra comptonii (S.Moore) Vink | E | 60 | 1 | 53 | 2.5 | 1279 |
| Sapotaceae | Pycnandra controversa (Guillaumin) Vink | E | 32 | 3 | 27 | 2.6 | 1298 |
| Sapotaceae | Pycnandra fastuosa (Baill.) Vink | E | 131 | 127 | 44 | 2.7 | 1073 |
| Sapotaceae | Pycnandra griseosepala Vink | E | 29 | 7 | 29 | 2.6 | 1103 |
| Sapotaceae | Pycnandra vieillardii (Baill.) Vink | E | 17 | 1 | 9 | 1.1 | 647 |
| Sapotaceae | Planchonella microphylla Pierre ex Dubard | E | 24 | 19 | 4 | 1.6 | 565 |
| Sapotaceae | Planchonella laetevirens (Baill.) Pierre ex Dubard | E | 43 | 42 | 24 | 1.9 | 1094 |
| Sapotaceae | Planchonella endlicheri (Montrouz.) Guillaumin | E | 171 | 156 | 46 | 2.4 | 954 |
| Sapotaceae | Planchonella wakere (Pancher & Sebert) Pierre | E | 99 | 89 | 17 | 2.2 | 751 |
| Sapotaceae | Planchonella lauracea (Baill.) Dubard | E | 67 | 63 | 30 | 3.3 | 1097 |
| Sapotaceae | Planchonella amieuana (Guillaumin) Aubrév. | E | 16 | 0 | 13 | 1.6 | 535 |
| Sapotaceae | Pycnandra cylindricarpa Swenson & Munzinger | E | 11 | 0 | 4 | 2.3 | 1141 |
| Sapotaceae | Pycnandra pubiflora Swenson & Munzinger | E | 31 | 31 | 7 | 1.9 | 722 |
| Sapotaceae | Pycnandra blaffartii Swenson & Munzinger | E | 5 | 0 | 5 | 0.3 | 444 |
| Sapotaceae | Planchonella sphaerocarpa (Baill.) Dubard | E | 21 | 2 | 13 | 2.3 | 525 |
| Sapotaceae | Planchonella rufocostata Munzinger & Swenson | E | 7 | 0 | 5 | 0.9 | 453 |
| Sapotaceae | Planchonella roseoloba Munzinger & Swenson | E | 9 | 0 | 8 | 0.8 | 651 |
| Sapotaceae | Planchonella glauca Swenson & Munzinger | E | 16 | 0 | 13 | 2.9 | 1189 |
| Sapotaceae | Pichonia dubia (Pierre ex Guillaumin) Swenson & Munzinger | E | 51 | 41 | 9 | 3.2 | 1287 |
| Sapotaceae | Pycnandra atrofusca Swenson & Munzinger | E | 13 | 13 | 2 | 1.6 | 460 |
| Sapotaceae | Planchonella latihila Munzinger & Swenson | E | 8 | 8 | 2 | 1.0 | 110 |
| Sapotaceae | Pycnandra gordoniifolia (S.Moore) Swenson & Munzinger | E | 32 | 13 | 10 | 1.8 | 624 |
| Sapotaceae | Pycnandra balansae (Baill.) Swenson & Munzinger | E | 95 | 31 | 59 | 2.8 | 833 |
| Sapotaceae | Pycnandra canaliculata Swenson & Munzinger | E | 27 | 27 | 3 | 1.9 | 822 |
| Sapotaceae | Pycnandra acuminata (Pierre ex Baill.) Swenson & Munzinger | E | 65 | 63 | 10 | 2.2 | 921 |
| Sapotaceae | Pycnandra sessiliflora Swenson & Munzinger | E | 38 | 36 | 10 | 1.6 | 739 |
| Sapotaceae | Pycnandra caeruleilatex Swenson & Munzinger | E | 4 | 4 | 0 | 0.2 | 298 |
| Sapotaceae | Pycnandra petiolata (Vink) Munzinger & Swenson, comb. ined. | E | 1 | 1 | 0 | 0.0 | 0 |
| Sapotaceae | Pycnandra deplanchei (Baill.) Swenson & Munzinger | E | 8 | 8 | 0 | 0.8 | 529 |
| Sapotaceae | Pycnandra sarlinii (Aubrév.) Swenson & Munzinger | E | 15 | 2 | 9 | 1.4 | 491 |
| Sapotaceae | Pycnandra confusa Swenson & Munzinger | E | 2 | 1 | 1 | 1.6 | 930 |
| Sapotaceae | Pleioluma balansana (Pierre ex Baill.) Swenson & Munzinger | E | 19 | 8 | 9 | 2.3 | 769 |
| Sapotaceae | Pleioluma baueri (Montrouz.) Swenson & Munzinger | E | 142 | 126 | 24 | 2.6 | 932 |
| Sapotaceae | Pleioluma lasiantha (Baill.) Swenson & Munzinger | E | 46 | 45 | 6 | 2.7 | 755 |
| Sapotaceae | Pleioluma novocaledonica (Dubard) Swenson & Munzinger | E | 10 | 10 | 1 | 1.2 | 800 |
| Sapotaceae | Pleioluma longipetiolata (Aubrév.) Swenson & Munzinger | E | 73 | 52 | 23 | 2.3 | 850 |
| Sapotaceae | Pleioluma sebertii (Pancher) Swenson & Munzinger | E | 74 | 74 | 7 | 1.8 | 557 |
| Sapotaceae | Pleioluma rubicunda (Pierre ex Baill.) Swenson & Munzinger | E | 62 | 13 | 49 | 2.6 | 1412 |
| Stemonuraceae | Gastrolepis austrocaledonica (Baill.) Tiegh. | E | 193 | 187 | 32 | 2.6 | 976 |
| Symplocaceae | Symplocos arborea (Vieill.) Brongn. & Gris | E | 59 | 8 | 38 | 2.3 | 759 |
| Symplocaceae | Symplocos caerulescens (Vieill.) Brongn. & Gris | E | 7 | 2 | 6 | 0.9 | 438 |
| Symplocaceae | Symplocos flavescens Brand | E | 57 | 45 | 22 | 1.7 | 884 |
| Symplocaceae | Symplocos montana (Vieill.) Brongn. & Gris | E | 205 | 116 | 112 | 3.2 | 1377 |
| Symplocaceae | Symplocos neocaledonica (Vieill.) Noot. | E | 82 | 2 | 53 | 2.7 | 1244 |
| Taxaceae | Austrotaxus spicata Compton | E | 38 | 4 | 31 | 2.4 | 1358 |
| Thymelaeaceae | Lethedon balansae (Baill.) Kosterm. | E | 59 | 38 | 46 | 2.6 | 1606 |
| Thymelaeaceae | Lethedon calleana (Guillaumin) Kosterm. | E | 11 | 10 | 4 | 3.1 | 1269 |
| Thymelaeaceae | Lethedon cernua (Baill.) Kosterm. | E | 40 | 38 | 15 | 3.0 | 1162 |
| Thymelaeaceae | Lethedon cordatoretusa Aymonin | E | 13 | 9 | 4 | 2.3 | 735 |
| Thymelaeaceae | Lethedon leratii (Guillaumin) Kosterm. | E | 23 | 18 | 7 | 3.6 | 1176 |
| Thymelaeaceae | Lethedon tannensis Sprengel | A | 21 | 11 | 3 | 2.2 | 943 |
| Thymelaeaceae | Solmsia calophylla Baill. | E | 188 | 175 | 24 | 3.2 | 992 |
| Trimeniaceae | Trimenia neocaledonica Baker f. | E | 33 | 0 | 25 | 2.7 | 1032 |
| Urticaceae | Dendrocnide latifolia (Gaudich.) Chew | A | 3 | 0 | 2 | 1.2 | 320 |
| Urticaceae | Dendrocnide peltata (Blume) Miq. | A | 1 | 0 | 1 | 0.0 | 0 |
| Violaceae | Hybanthus micranthus Guillaumin | E | 9 | 4 | 2 | 1.0 | 514 |
| Winteraceae | Zygogynum acsmithii Vink | E | 14 | 13 | 4 | 1.1 | 1241 |
| Winteraceae | Zygogynum amplexicaule (Vieill. ex P.Parm.) Vink | E | 65 | 6 | 51 | 2.7 | 1286 |
| Winteraceae | Zygogynum baillonii Tiegh. | E | 40 | 39 | 18 | 2.5 | 1320 |
| Winteraceae | Zygogynum bicolor Tiegh. | E | 11 | 2 | 11 | 0.9 | 412 |
| Winteraceae | Zygogynum comptonii (Baker f.) Vink | E | 4 | 1 | 3 | 1.9 | 365 |
| Winteraceae | Zygogynum crassifolium (Baill.) Vink | E | 46 | 46 | 9 | 2.0 | 1058 |
| Winteraceae | Zygogynum mackeei Vink | E | 3 | 1 | 3 | 1.9 | 329 |
| Winteraceae | Zygogynum pancheri (Baill.) Vink | E | 111 | 71 | 58 | 2.2 | 1286 |
| Winteraceae | Zygogynum pauciflorum (Baker f.) Vink | E | 12 | 0 | 12 | 0.7 | 827 |
| Winteraceae | Zygogynum pomiferum Baill. | E | 80 | 38 | 50 | 2.7 | 1179 |
| Winteraceae | Zygogynum stipitatum Baill. | E | 38 | 2 | 34 | 2.3 | 1510 |
| Winteraceae | Zygogynum tieghemii Vink | E | 34 | 29 | 22 | 2.9 | 1314 |
| Winteraceae | Zygogynum vinkii Sampson | E | 10 | 0 | 9 | 2.2 | 484 |
