# Supplementary material for: Environmental correlates for tree occurrences, species distribution and richness on a high-elevation tropical island
Source: AoB Plants. 2015 Jul 10;7:plv075. doi: 10.1093/aobpla/plv075 (PMC4561634; doi:10.1093/aobpla/plv075)
Supplement: Additional Information [file supp_plv075_plv075supp_file2.doc]

File 2. Simplified list of reports related to inventory studies and surveys in New Caledonian forests that have most contributed to the tree occurrences dataset “observations”

Barrabé L, Rigault F, Dagostini G, Munzinger J. 2007. Recensement du patrimoine botanique des aires protégées terrestres de la Province Sud - synthèse bibliographique. Nouméa : Rapport intermédiaire convention IRD/ DRN-PS N°125-06, 88p.

Barrabé L, Rigault F, Dagostini G, Nigote W, Munzinger J. 2008. Recensement du patrimoine botanique des aires protégées terrestres de la province sud - Caractérisation et cartographie des formations végétales de 10 aires protégées terrestres de la province sud. Nouméa: Rapport de convention IRD/DENV-PS N°C-193-07 -CD2006-2010, 110p.

Barrière R, Dagostini G, Rigault F, Munzinger J. 2007. Caractérisation et cartographie de la végétation des Milieux miniers de la côte nord-ouest. Nouméa: Rapport de Convention IRD/DDEE Province Nord, N°32/2007, 62p.

Barrière R, Nigote W, Rigault F, Dagostini G, Munzinger J. 2008. Caractérisation et cartographie des végétations des milieux ultramafiques de la côte nord-est de la Province nord. Nouméa: Rapport de Convention IRD/DDEE Province Nord, N°162/2006.

Birnbaum Ph, et Mangeas M. 2014. Projet CoRiFor: Caractérisation des connectivités structurelle et fonctionnelle des paysages fragmentés sur sols ultramafiques. Rapport intermédiaire CNRT/IAC/CIRAD/IRD, 35p.

Birnbaum Ph, Hequet V, Vandrot H, Ibanez T, et Blanchard E. 2013. Cartographie et caractérisation des faciès forestiers sur sols volcano-sédimentaires en Province Nord de Nouvelle-Calédonie. Rapport final. Convention AMAP/Province Nord/IAC/IRD/CIRAD, N°10C113, 49 p.

Dagostini G, Rigault F, Munzinger J. 2008. Inventaire floristique des groupements végétaux du massif de Poum. Convention IRD/ SLN, 29p.

Dagostini G. 2006. Inventaires floristiques de différentes stations du nord sur sols serpentinisés. Recherche de sites comparatifs à la presqu’île de Vavouto. Nouméa: Rapport de Mission IRD/FalconBridge. 14p.

Dagostini G. 2012. Cartographie des types de végétation et inventaires floristiques de la zone de conservation de la Wadjana. Nouméa: Rapport de convention IRD/Goro, N°2417, 68p.

Grignon C. 2006. Accumulation et synthèse de données floristiques relatives à la réserve de la "forêt nord" de Nouvelle-Calédonie. Paris : Master 2, Université Pierre & Marie Curie, 63p.

Grignon C, Dagostini G, Rigault F, Munzinger J. 2009. Recensement du patrimoine botanique des aires protégées terrestres de la province sud - Caractérisation et cartographie des formations végétales de 8 aires protégées terrestres de la province sud. Nouméa : Rapport d’étapes 2 – Convention IRD/DENV-PS, N°C-193-07 -CD2006-2010, 130p.

Grignon C, Rigault F, Dagostini G, Munzinger J. 2010. Recensement du patrimoine botanique des aires protégées terrestres de la province sud - Caractérisation et cartographie des formations végétales de 4 aires protégées terrestres de la province sud. Nouméa: Rapport d’étapes 3 – Convention IRD/DENV-PS, N° C-193-07 -CD2006-2010, 80p.

Grignon C, Chambrey C, Rigault F, Munzinger J. 2011. Recensement du patrimoine botanique des aires protégées terrestres de la province sud - Caractérisation et cartographie des formations végétales de 3 aires protégées terrestres de la province sud. Nouméa: Rapport d’étapes 4 - Convention IRD/DENV-PS, N° C-193-07 -CD2006-2010, 72p.

Ibanez T, et Birnbaum Ph. 2012. Dynamique des habitats forestiers en Nouvelle-Calédonie. Nouméa : Rapport d’avancement. Convention CIRAD/IAC/DENV-PS N° C 315-12, 16p.

Jaffré T. 1988. Végétation et flore de la Chute de la Madeleine, Nouvelle-Calédonie. Nouméa: ORSTOM. 11p

Jaffré T, Dagostini G, Rigault F. 2003. Identification typologie et cartographie des groupements végétaux de basse altitude du grand sud calédonien et de la vallée de la Tontouta. Caractérisation botanique et écologique des écosystèmes représentatifs. Nouméa : Convention IRD / Province Sud n°6024-12-2000 / DRN-ENV. 43p + annexes.

Jaffré T, Fambart-Tinel J, Roumagnac F. 2003. Reconnaissance et pré-caractérisation de la végétation et de la flore de la presqu’île de Vavouto, des zones forestières qui seront touchées par l’édification d’un barrage sur la Pouembout et du massif du Koniambo. Nouméa : rapport de convention IRD/Falconbridge, 10p+annexes.

Jaffré T, Dagostini G, Rigault F. Coic N. 2004. Inventaire floristique des unités de végétation de la zone d’implantation des infrastructures minières et industrielles de Goro Nickel. Nouméa : Rapport final de convention IRD/Goro-Nickel- Milieu écologique terrestre / Flore – Annexe III-A-5-4. 42p + annexes.

Jaffré T, Roumagnac F, Fambart-Tinel J. 2004. Analyse de la flore du premier secteur d'exploitation minière sur le massif du Koniambo. Convention IRD/Falconbridge, 5p + annexes.

Juge R, Kaqea N, Le Borgne T, McCoy S. 2006. Les inventaires floristiques des formations végétales de la future pépinière et de l'extension du camp de la géologie à grand lac. Goro-Nickel, 12p.

McCoy S 2006. L’inventaire floristique des maquis para-forestiers dans l’emprise des bassins : eaux de ruissellement du stockage de souffre et de premier flot. Goro-Nickel-Milieu écologique terrestre / Flore – Annexe III-A-5-7, 21p.

Munzinger J, Dagostini G, Rigault F, Jaffré T. 2004. Inventaire floristique des groupements végétaux des nouveaux tracés proposés par Goro Nickel SA pour le passage de l'émissaire à Port Boisé. Nouméa: Rapport d’expertise IRD/Goro-Nickel, 14p.

Munzinger J, Dagostini G, Rigault F. 2005. Inventaire floristique de la concession minière Byzance Red, située sur le massif du Koungouhaou Nord. Nouméa : Rapport d’expertise IRD/AIME-NC. 23p.

Munzinger J, Dagostini G, Rigault F, Kurpisz D. 2007. Inventaire de la réserve de la Forêt Nord. Nouméa : Rapport de consultance IRD/Goro-Nickel SA. 52p.

Munzinger J, McPherson G, Lowry II PP. 2007. Results from the Inventory of the Kouakoué Massif, New Caledonia. Progress Report for National Geographic Society Grant # 7579-04, 14p

Munzinger J, Kurpisz D, Rigault F, Dagostini G. 2008. Caractérisation taxonomique et patrimoniale des lambeaux forestiers dans le grand sud calédonien, Implication pour la gestion et la préservation de ces formations – Nouméa : Rapport de Convention IRD/DRN Province Sud, N°6024-60/2005, 74p.

Munzinger J, Birnbaum P, Butin J-P, Callmander M, Hequet V, Lowry II PP, Vandrot H. 2011. Rapport préliminaire - RAP dans la massif du Mont Panié, Nouvelle-Calédonie. Nouméa: Institut de recherche pour le Développement. 18p.

Munzinger J. 2013. Inventaire botanique du massif du Panié et des roches de la Ouaième, Province Nord, Nouvelle-Calédonie. In: Tron F, Franquet R, Larsen TH, Cassan J-J, eds. *Evaluation rapide de la biodiversité du massif du Panié et des Roches de la Ouaième, Province nord, Nouvelle-Calédonie. RAP Bulletin of Biological Assessment*. Arlington, VA, USA: Conservation International.

Pillon Y. 2009. Compte-rendu de la mission « Côte oubliée » du 10 au 14 novembre 2008 : Inventaire floristique préliminaire des vallées de la Ni et de la Pourina. IRD, 11p.

Rigault F, Dagostini G, Jaffré T. 2000. Caractérisation des groupements végétaux et de la flore des trois périmètres miniers de la région de Mamié. Nouméa: Rapport de consultance ORSTOM/ Queensland Mining, 10p + annexes.

Spir I. 2005. Synthèse bibliographique et projet de recherche : Végétation des contreforts Est de la réserve spéciale botanique du Mont Panié, valeur patrimoniale des formations rencontrées, identification des menaces et proposition de mesures de conservation. Orléans: Master 2 ETAH, Université d’Orléans, 14p.
